# Fire blight resistance, irrigation and conducive wet weather improve *Erwinia amylovora* winter survival in cankers

**DOI:** 10.3389/fmicb.2022.1009364

**Published:** 2022-10-18

**Authors:** Ricardo D. Santander, Fatemeh Khodadadi, Christopher L. Meredith, Željko Rađenović, Jon Clements, Srđan G. Aćimović

**Affiliations:** ^1^Irrigated Agriculture Research and Extension Center, College of Agricultural, Human, and Natural Resource Sciences, Washington State University, Prosser, WA, United States; ^2^Hudson Valley Research Laboratory, School of Integrative Plant Science, Plant Pathology and Plant-Microbe Biology Section, Cornell University, Highland, NY, United States; ^3^Alson H. Smith Jr. Agricultural Research and Extension Center, School of Plant and Environmental Sciences, Virginia Polytechnic Institute and State University, Winchester, VA, United States; ^4^Center for Agriculture, Food, and the Environment, University of Massachusetts Amherst, UMass Cold Spring Orchard, Belchertown, MA, United States

**Keywords:** viability dPCR, propidium monoazide, pathogen detection, irrigation, symptom severity, pathogenicity related proteins, gene expression, pathogen population dynamics

## Abstract

*Erwinia amylovora* causes fire blight, a disease responsible for enormous economic losses in the pome fruit-producing areas where it is present. Despite the abundant research on fire blight, information about *E. amylovora* population dynamics and survival in fire blight cankers and the plant defense responses to this pathogen in the infected bark are limited. In our study, we obtained fire blight cankers in apple, pear, and Asian pear cultivars showing differing resistance to the disease by shoot inoculation with *E. amylovora*. We collected cankers from irrigated and non-irrigated trees every 3 months in two independent field experiments and analyzed samples by viability digital PCR. We also assessed the expression of pathogenicity-related (*PR*) genes in the bark of selected apple and Asian pear cultivars. A logistic regression analysis revealed the impact of environmental and host factors on *E. amylovora* detection rates in cankers. The chances of detecting live *E. amylovora* cells in cankers increased significantly in those collected from irrigated trees, in July, and/or during an experiment performed in a year with an expected average rainfall when compared to samples from non-irrigated trees, collected in January, and/or during an experiment performed under environmental conditions dominated by drought. We found a positive correlation between the pathogen detection rates in cankers and the host resistance to fire blight that might be explained by lower *E. amylovora* survival rates in more damaged tissues of susceptible hosts. The genes *PR-1, PR-2, PR-5,* and *PR-8* were induced in the bark surrounding apple and Asian pear fire blight cankers. Our study, involving the analysis of more than 800 canker samples, provides new knowledge about the fire blight disease cycle and lays the foundation for improved fire blight management and eradication strategies in pome fruit orchards.

## Introduction

Fire blight of rosaceous plants is caused by the bacterial pathogen *Erwinia amylovora* (Burrill; Winslow et al., 1920). The disease affects around 180 plant species, including economically important fruit crops like apple, pear, and Asian pear, as well as ornamental and wild plant species (EPPO, 2022). Since initially reported in 1780 on pear and quince in the Hudson River Valley in New York (United States; Denning, 1794), fire blight has spread worldwide to more than 50 countries (CABI, 2022; EPPO, 2022), posing a serious economic threat to the pome fruit production regions where the pathogen is present (Longstroth, 2001; Hasler et al., 2002; Norelli et al., 2003).

The main symptoms associated with fire blight include necrosis and ooze droplets, and the most susceptible plant organs are flowers, young leaves, actively growing shoots, and immature fruit. As the infection progresses, *E. amylovora* cells from flowers and/or green tissues reach lignified organs. The invasion of the perennial bark of branches, trunk, rootstock, and occasionally roots, by the pathogen usually leads to the development of fire blight cankers (Bogs et al., 1998; Thomson, 2000; van der Zwet et al., 2012). During this process, the host cells of the cortical parenchyma adjacent to the infected area differentiate into a defense periderm. This new layer of tissue surrounds the pathogen, forming a suberized barrier lacking intercellular spaces that blocks *E. amylovora*’s access to healthy plant tissues. Depending on the degree of periderm development around the diseased area, cankers are visually classified into two types: determinate (cracked margin separates the infected area from healthy tissues) and indeterminate (diffuse margin between diseased and healthy tissues; Schouten, 1993; Biggs, 1994; Thomson, 2000;). However, cankers are rarely entirely determinate or indeterminate and often possess a combination of the two types of margins (Beer and Norelli, 1977).

The importance of cankers in fire blight disease cycle is in their role reservoirs and inoculum sources of *E. amylovora*. The pathogen overwinters in some cankers (Thomson, 2000). With the host’s growth renewal in spring, bacterial ooze droplets can emerge on the surface of active cankers in the orchard. These droplets are composed of *E. amylovora* cells in a matrix of bacterial exopolysaccharides (EPS) and plant sap (Slack et al., 2017). Insects, rain, wind and contaminated pruning tools can transmit the pathogen from diseased plants to healthy parts of the same or other plants (Thomson, 2000; Ordax et al., 2015; Slack et al., 2017; Boucher et al., 2021). Apart from the contribution of oozing cankers to the external spread of *E. amylovora,* cankers have also been associated with the internal transmission of the pathogen to shoots and tissues close to cankers (Hickey et al., 1999; Thomson, 2000; van der Zwet et al., 2012). Although different authors have described actively oozing cankers being usually of the indeterminate type, *E. amylovora* is also present on the surface of many apparently inactive cankers with determinate margins (Miller and Schroth, 1972; Schroth et al., 1974; Thomson et al., 1975; Beer and Norelli, 1977). To date, the role of determinate cankers in fire blight epidemics has not been elucidated.

Despite the abundant works on fire blight, there is limited information about the actual *E. amylovora* population sizes in cankers, their dynamics over time, and the effects of external and host-related factors impacting the pathogen numbers. Most studies addressing *E. amylovora* survival or its detection in cankers have used classical microbiology methods and/or regular PCR to detect this bacterium, mainly on the canker surface (Miller and Schroth, 1972; Beer and Opgenorth, 1976; Beer and Norelli, 1977; Beer, 1978; Paulin, 1981; Sobiczewski et al., 1999; Aćimović et al., 2014). However, there are important drawbacks associated with both culture-dependent and PCR-based detection methods. *E. amylovora* cells in cankers are exposed to nutritional stress, saprophytic microbiota, suboptimal temperatures, and other challenges which have been related to the loss of the pathogen capacity to form colonies on solid media while remaining viable (Ordax et al., 2006; Biosca et al., 2008; Santander et al., 2014; Santander and Biosca, 2017). The use of selective media for *E. amylovora* detection may also contribute to underestimating of the actual pathogen cell numbers in cankers due to the detrimental effect of the selective compounds on stressed cells in comparison to the non-stressed ones (Apajalahti et al., 2003; Özkanca et al., 2009). On the other hand, molecular detection of the pathogen by classic PCR does not discriminate live from dead cells. This technique only provides qualitative data, and often, PCR-based detection results vary in comparison to those using culture-dependent methods (Sobiczewski et al., 1999, 2006, 2017; Kielak et al., 2002; Ordax et al., 2015). To address the inconveniences of both culture media and PCR-based detection methods, in this work we used a previously validated protocol for canker sample processing and viability digital PCR (v-dPCR) analysis to monitor *E. amylovora* populations in cankers. The advantages of this methodology are the selective detection and accurate quantification of live *E. amylovora* cells in cankers without relying on calibration curves, while maintaining a good correlation with culturability data (Santander et al., 2019, 2022).

Another barely explored aspect of fire blight is the host’s genetic response to the pathogen in lignified bark tissues. Several studies have demonstrated the induction of pathogenicity-related (*PR*) genes in apple shoots, leaves, and flowers during infections with *E. amylovora* (Venisse et al., 2002; Bonasera et al., 2006; Milčevičová et al., 2010; Hassani et al., 2016), and have evidenced a link between the induction of *PR* genes and enhanced resistance of apple tissues against fire blight (Maxson-Stein et al., 2002; Malnoy et al., 2007; Aćimović et al., 2015). Some of the previously studied genes and their relationship with the systemic acquired resistance (SAR) against fire blight in apple encode the PR proteins PR-1 (antifungal properties), PR-2 (β-1,3-glucanase), PR-5 (thaumatin-like protein) and PR-8 (class III chitinase). However, there is no information about the expression of *PR* genes in the bark near fire blight cankers or if these responses vary depending on the host species and their susceptibility to fire blight.

Through the v-dPCR analysis of more than 800 cankers from different apple, pear and Asian pear cultivars, in two independent experiments, the main goals of this study were to quantify *E. amylovora* populations in cankers over time and explore the parameters conditioning *E. amylovora* detection in cankers. Besides, we analyzed potential correlations between *E. amylovora* cell concentrations in cankers, the host resistance to fire blight, and the pathogen detections after winter. Finally, we explored the expression of *PR* genes in the bark surrounding fire blight cankers and analyzed possible differences in *PR* gene expression patterns linked to the *E. amylovora* host species and tree irrigation.

## Materials and methods

### Environmental conditions during the field experiments

The weather conditions in the locations where the experiments were performed (Highland, NY, and Belchertown, MA) were retrieved from the SC-ACIS (Applied Climate Information System) web service^1^ (DeGaetano et al., 2015), and from the National Integrated Drought Information System^2^ (Svoboda et al., 2002). The former provides access to climate data from daily U.S. weather stations in the Global Historical Climate Network and other data networks. The latter shows the geographical location and intensity of drought across the U.S. at national, tribal, state, and local levels. The drought categories in this service are based on the assessments of different parameters linked to dryness and drought, like the available water in streams, lakes, and soils compared to the usual conditions reported for the same time of the year.

### Plant material and irrigation treatments

The fluctuation of live *E. amylovora* populations in fire blight cankers collected from July until April of the next year was analyzed in apple (*Malus pumila* Mill.), pear (*Pyrus communis* L.) and Asian pear (*P. pyrifolia* Nakai). To develop cankers, we shoot-inoculated the apple cultivars ‘Cortland’ (clone Royal Court), ‘Cameo’, ‘Honeycrisp’ and ‘Red Delicious’; pear cultivars ‘Bartlett’ and ‘Bosc’; and Asian pear cultivars ‘Hosui’, ‘Kosui’, ‘Olympic’, ‘Shinsui’, ‘Shinko’, ‘Ya-Li’ and ‘Yoinashi’. However, after an initial assessment of the success of shoot inoculations and canker formation, the apple and Asian pear cultivars ‘Red Delicious’ and ‘Olympic’, respectively, were removed from the main experiment because of the low success of infection and canker formation. The Asian pear cultivars ‘Kosui’, ‘Shinsui’ and ‘Ya-Li’ were also removed from the experiment due to the lack of trees of the same cultivars to repeat the experiment. All the cultivars listed above, including the eliminated ones, were used to determine the fire blight susceptibility index.

All the apple trees used in this work were planted in May 2001 at the Hudson Valley Research Laboratory (HVRL) orchards of Cornell AgriTech, in Highland, NY (United States). In addition to assessing *E. amylovora* populations in cankers, the apple trees were used to study the effect of irrigation on the populations in cankers. For this aim, the irrigation lines of a group of apple trees of each cultivar were disconnected from the irrigation system from June to September, so that the only water the trees received was from rain events. A control group of apple trees remained connected to the irrigation system throughout the same period. The irrigation system consisted of drip emitters spaced 3 ft. (0.91 m) apart, working from 12 pm to 4 pm daily, each putting out 0.6 gal (2.27 l) per hour. In total, the irrigated tree block area (1,821 m^2^) received around 1.36 l/m^2^ daily. Experiments with apple trees were performed in 2016–2017 and repeated in 2018–2019 in the same location. In both experimental repeats, a Flow32A-1 K sap flow sensor (Dynamax, Houston, United States) was used to compare the sap flow rates in irrigated and non-irrigated trees.

Regarding pear and Asian pear trees, the first repeat of the experiment (2016–2017) was performed at the same time and location as apples, using trees planted in 2010–2011. After this experiment, most pear and Asian pear trees were severely damaged or died due to fire blight. To repeat the experiment, in 2017–2018, we used pear and Asian pear trees of similar age of the cultivars ‘Bosc’ and ‘Bartlett’, and ‘Hosui’, ‘Shinko’ and ‘Yoinashi’, respectively, located at the UMass Cold Spring Orchard in Belchertown, MA. None of the *Pyrus* spp. trees in Highland, NY (first experimental repeat) and Belchertown, MA (second experimental repeat) were connected to irrigation system.

### Shoot inoculations

Cankers were obtained by shoot inoculation with the American *E. amylovora* strain Ea273 (ATCC 49946). Bacterial inocula were prepared using overnight cultures in LB at 28°C (180 rpm). After washing cells twice with 10 mM phosphate buffered saline (PBS) pH 7.0, cell suspensions were adjusted to 1 × 10^9^ CFU/ml in PBS and refrigerated in a polystyrene container with ice until use.

Inoculations were carried out during the last 10 days of June in all the experimental repeats, on newly developed shoots from perennial branches, before terminal bud set, when shoot lengths reached between 5 and 7.5 inches (13–19 cm). Each shoot was cut diagonally, just below the tip, with a surface-disinfected scalpel to create a sleeve cut on the stem. Then, 40 μl of the bacterial suspension were placed between the exposed tissues and the cut sleeve (around 4 × 10^7^ CFU/shoot) with a micropipette, allowing the plant tissue to absorb the inoculum droplet within the next minutes (Supplementary Figure S1).

Because cankers do not always develop after shoot inoculation, 35 shoots per tree, host cultivar, and irrigation treatment were inoculated to ensure a minimum number of cankers developed per tree. Inoculations were repeated in three to five tree replicates per host cultivar and irrigation treatment. Non-inoculated trees were used as negative controls during sample analysis.

### Collection, storing, processing, and PMAxx treatment of canker samples

To assess the effect of the host species, cultivar, irrigation treatment and the year period on *E. amylovora* populations in cankers, at least three canker samples were collected per replicate tree in Summer (within the last 10 days of July), fall (last 10 days of October), winter (last 10 days of January) and spring (last 10 days of April). During the first experimental repeat, right before collecting the first canker samples in July, the number of infected shoots and cankers formed was recorded to estimate the incidence of infections after shoot inoculation.

Samples were collected using surface disinfected pruning shears by cutting the piece of the branch containing the canker, about 2.5 cm above and below the canker margin, according to Santander et al. (2019, 2022). Then, samples were placed into hermetically sealed plastic bags and immediately fast-frozen in an all-plastic Dewar flask filled with enough liquid nitrogen to cover the samples. The frozen samples were stored at-80°C until use.

For sample processing, the canker length was measured with a caliper. Then, an area including the entire canker surface plus 2 mm of bark around the canker margin was delimited with a sterile scalpel, and canker tissues within this area, including the bark and the vascular cambium, were aseptically excised, cut into pieces, weighted, and placed into a plastic bag containing ice-cold 0.1x AMB (Antioxidant Maceration Buffer, EPPO, 2022) in a ratio of 50 ml of 0.1x AMB per gram of canker. The prepared samples were then homogenized in the plastic bag by repeated hammering against a hard surface and placed on ice for up to 30 min until use, as described previously (Santander et al., 2019, 2022).

To selectively quantify live *E. amylovora* cells in cankers, we used a previously optimized protocol for this type of samples using propidium monoazide (PMA; Santander et al., 2019, 2022). Briefly, plant macerates were treated with 100 μM PMAxx (Biotium Inc., CA, United States) and 1x PMA Enhancer for Gram Negative bacteria (Biotium Inc., CA, United States; final concentrations) for 5 min. Then, PMAxx was photo-activated for 10 min by light exposure using two 500 W halogen bulbs while incubating on ice. The treated sample macerates were pelleted by centrifugation and stored at-80°C until use.

### DNA extraction and viability dPCR conditions

The DNA from the pelleted PMAxx-treated samples was extracted using the DNeasy Plant Mini Kit (Qiagen, Hilden, Germany) following the manufacturer’s instructions, eluting DNA in a final volume of 200 μl.

The chip-based QuantStudio 3D (QS3D) dPCR System (ThermoFisher Scientific, Waltham, MA, United States) was used to detect and quantify live *E. amylovora* cells in canker samples, according to Santander et al. (2019, 2022). Briefly, each dPCR reaction mix contained 8 μl of 2x QS3D dPCR Master Mix v2 (Applied Biosystems, Frederick, MD, United States), 0.8 μl of 20x primers/probe mix containing the primers Ams06KbF (Santander et al., 2019) and Ams189R (Pirc et al., 2009) and the probe Ams141T (Pirc et al., 2009; final concentration of primers and probe in the master mix, 0.9 μM and 0.2 μM, respectively) plus 6.4 μl of DNA sample and 0.8 μl of nuclease-free water, in a final volume of 16 μl. The QS3D dPCR 20 K chips v2 were loaded with 15 μl of the reaction mixture, and the dPCR amplification was conducted in a GeneAmp 9,700 PCR thermocycler (Applied Biosciences, Foster City, CA, United States). The thermal cycling consisted of 10 min at 95°C for DNA polymerase activation, followed by 39 two-step cycles of 1 min at 60°C and 15 s at 98°C, followed by a final extension at 60°C for 2 min.

After DNA amplification, chips were read with the QS3D Instrument for Imaging. Quantitative data were analyzed with the QS3D AnalysisSuite Cloud Software (version 3.1.2-PRC-build-03, Thermo Fisher Scientific, Waltham, MA, United States). The original target DNA (i.e., live *E. amylovora* cell) concentration per gram of canker was calculated using the copies of target DNA per μL provided by the QS3D AnalysisSuite Cloud Software, taking into consideration the applied dilution factors, sample volumes and DNA extraction efficiencies for each type of plant material analyzed (Santander et al., 2019). Only the chips with more than 15,000 partitions qualifying for quantification were used for analysis. No-template controls (dPCR reaction mix with water instead of template DNA) and DNA extractions from *E. amylovora*-free plant material (from each apple, pear, and Asian pear cultivar included in the study), as well as positive controls artificially inoculated with different *E. amylovora* concentrations, were used in preliminary assays to discriminate background fluorescence from positive calls, as described by Santander et al. (2019). We considered samples positive for *E. amylovora* detection when the calculated copies/mL reached values equal or above the lower detection limit (around 5 × 10^4^ copies/g of canker).

### Rating of host cultivar resistance to fire blight

The fire blight resistance of different host plant species and cultivars may vary depending on the growing, environmental and tree grafting conditions, the *E. amylovora* strain causing the infection, and the host species and cultivars compared in each case. This explains the relatively variable degree of fire blight resistance reported previously for the same apple, pear and Asian pear cultivars (Supplementary Table S1). To analyze potential correlations between the host resistance to fire blight and other variables, we classified each cultivar as resistant, moderately resistant, moderately susceptible, susceptible, highly susceptible and extremely susceptible, by combining two indexes quantifying (i) the severity of symptoms observed in the field and (ii) the incidence of fire blight symptoms in each cultivar, respectively.

The severity of fire blight symptoms (shoot blight onset and progression) of each cultivar in the field was assessed after shoot inoculation. We rated each cultivar from more resistant to more susceptible using a Necrosis Severity Index (NSI), where NSI = 0 means absence of fire blight symptoms; NSI = 1, very slow or no symptom progression after the onset of symptoms, and extremely rare death of branches in current year; NSI = 2, slow symptom progression and only occasional death of branches in current year; NSI = 3, faster symptom progression in comparison to cultivars classified as NSI = 2, with usual death of a small number of branches with cankers in current year; NSI = 4, faster symptom development in comparison to trees classified as NSI = 3, and frequent death of branches with cankers in current year; SSI = 5, the fastest symptom development in comparison to NSI = 1–4, and widespread death of branches or the whole tree in current year.

To determine the average incidence of fire blight symptoms per tree, we used the percent of cankers formed in relation to the number of blighted inoculated shoots (% C/S) as a measure of the symptom incidence for each tree. We named this index the Effective Canker Formation Index (ECFI). We used the % C/S per tree, and not the percentage of symptomatic shoots per tree (% S) or the percentage of formed cankers per tree (% C) as a measure of symptom incidence, because % C/S links both parameters, and because the preliminary analysis indicated a better correlation between classifications performed using the NSI and the % C/S, than using the % S or the % C. Similar to the NSI, we rated the cultivar susceptibility to fire blight with an ECFI ranging from 0 to 5 (from less to more susceptible) based on the average % C/S calculated for each cultivar, as follows: % C/S = 0%, ECFI = 0; % C/S = (1–20%), ECFI = 1; % C/S = (21–40%), ECFI = 2; % C/S = (41–60%), ECFI = 3; % C/S = (61–80%), ECFI = 4; % C/S = (81–100%), ECFI = 5.

With the obtained NSI and ECFI for each cultivar, we created a Fire Blight Susceptibility Index (FBSI) by combining (summing) the results of both indices, and classifying each cultivar accordingly as follows, FBSI = 0, resistant (R); FBSI = [1, 2], moderately resistant (MR); FBSI = [3, 4], moderately susceptible (MS); FBSI = [5, 6], susceptible (S); FBSI = [7, 8], highly susceptible (HS); FBSI = [9, 10], extremely susceptible (ES).

This information was used to study potential correlations between the host resistance and the *E. amylovora* population sizes in cankers and its detection rates throughout the year.

### RNA isolation, cDNA synthesis, and real-time PCR conditions

To investigate the expression patterns of different *PR* genes in the bark near cankers on hosts with different levels of resistance to fire blight, we used canker samples from the apple cultivars ‘Honeycrisp’ (moderately susceptible) and ‘Cortland’ (highly susceptible) and the Asian pear cultivars ‘Hosui’ (extremely susceptible) and ‘Shinko’ (highly susceptible). We also assessed potential differences between *PR* gene expression in apple cankers from irrigated and non-irrigated trees. All the cankers used in the expression analysis were collected in July, 1 month after shoot inoculation. For each cultivar and/or irrigation treatment, we analyzed cankers from three different replicate trees (three cankers per replicate tree). Healthy bark tissues from branches in non-inoculated trees were used as negative controls.

For RNA extraction, canker samples stored at -80°C were processed while frozen. Pieces of bark surrounding the cankers were removed from tissues with a sterile hollow wood punch borer (9 mm diameter) and powdered in an ice-cold mortar using liquid nitrogen and pestle. Powdered samples were stored at -80°C until use. For RNA extraction, different commercial column-based kits were tested. We obtained better RNA quality and yields for apple cankers using the E.Z.N.A.® Fungal RNA Mini Kit (Omega Bio-Tek, Frederick, Colorado, United States). In the case of Asian pear cankers, the only kit allowing an acceptable RNA extraction from bark tissues was the Spectrum Plant Total RNA Kit (Sigma-Aldrich, St. Louis, MO, United States). Total RNAs were treated with DNAse I (Ambion, Austin, TX, United States) to remove possible genomic DNA fragments co-extracted with RNA. The purity and quantity of the extracted RNA were measured spectrophotometrically with a NanoDrop, and RNA integrity was checked on agarose gels. Samples showing poor RNA quality were removed from the experiment. To obtain cDNA, 200 ng of each RNA sample were reverse transcribed with the High-Capacity cDNA Synthesis Kit (Applied Biosystems, Foster City, CA) following the manufacturer’s protocol.

For the expression analysis, we used primers for the genes *PR-1, PR-2, PR-5, PR-8* and the actin reference gene published previously (Supplementary Table S2; Maxson-Stein et al., 2002; Bonasera et al., 2006). The real-time PCR reactions were prepared at a final volume of 20 μl, containing (final concentrations) 1x Maxima SYBR Green qPCR Master Mix (Thermo Fisher Scientific, Waltham, MA, United States), 20 ng sample cDNA, and primers at 0.3 mM each. The thermal cycling was performed in a LightCycler 480 II System (Roche Diagnostics, Indianapolis, IN, United States) using 96-well plates. For every sample, a set of triplicate reactions for the relative quantification of *PR-1*, *PR-2*, *PR-5*, *PR-8* and actin gene expression, plus negative controls without template DNA, were included in the same plate. The real-time PCR cycling conditions consisted of a 5 min initial denaturation at 95°C, followed by 40 cycles of 15 s of denaturation at 95°C, 30 s of annealing at 60°C, and 30 s of extension at 72°C. Melting curves were obtained after 40 cycles by a denaturation step at 95°C for 5 s, followed by annealing at 65°C for 1 min and 97°C with a heating rate of 0.1°C/s and continuous fluorescence measurement. Final cooling was performed at 40°C for 30 s.

The changes in gene expression were calculated relative to the reference gene actin by the Livak-Scmittgen (2^-ΔΔCt^) method (Livak and Schmittgen, 2001), using the basal expression in negative control samples (healthy bark tissue) as a calibrator.

### Statistical analysis

The association between *E. amylovora* detection in cankers and variables such as the irrigation treatment, host species/cultivar, canker harvesting season, host resistance to fire blight, etc., was analyzed by logistic regression. The relationships between individual variables and *E. amylovora* detection were first assessed by a univariate approach. Then, the simultaneous effect of all the variables that showed significant association with *E. amylovora* detection was modeled by multivariable logistic regression. The logistic model that was more likely to generate the data was selected after pairwise comparisons between the more complex model containing all the variables and simpler models lacking one or more variables. Criteria for selecting the best models were based on differences between the model’s AICc values (Akaike’s Information Criterion corrected for sample size) and estimators of the model’s capacity to predict the obtained results, using different goodness-of-fit metrics, including classification methods, pseudo *R*^2^ values and hypothesis tests. The potential multicollinearity between two or more variables in the final model was evaluated in terms of the variance inflation factor (VIF).

For the analysis of the *E. amylovora* cell concentrations in cankers and canker sizes, diagnostics tests indicated the non-Gaussian distribution of the residuals, in many cases, even after the log transformation of data. Hence, comparisons between data sets were performed by non-parametric tests. Differences between two groups of samples were assessed with unpaired two-tailed Mann–Whitney *U* tests, and between three or more groups of samples, by Kruskal-Wallis ANOVA followed by Dunn’s multiple comparisons tests. In figures and in the text, to avoid the effect of extremely high and/or low values, we used the median instead of the mean as a measure of central tendency and interquartile ranges (IQR) instead of the standard deviation as estimators of the data dispersion.

Correlation analyses were performed by pairing individual values for each variable. In each analysis, Pearson’s correlation coefficient (r) was used as an indicator of the strength and sign of the correlation. The coefficient of determination (*r*^2^) was also calculated to estimate the proportion of the variation of one factor potentially caused by its relationship to the other factor. The *p* values indicating the significance of the correlation were used as a criterium to determine the chances that random sampling would result in a correlation coefficient as low/high as the one observed in the experiment.

For the analysis of real-time PCR data, relative expression values were normalized by log-transformation, and differences between the expression of *PR* genes in control samples and cankers were assessed by two-tailed unpaired *t-*tests.

In all cases, *p* values <0.05 were considered statistically significant. All the statistical analyses in this work were carried out using GraphPad Prism 9 (version 9.3.1) for macOs.

## Results

### Environmental conditions during the experiments

**Figure 1:**
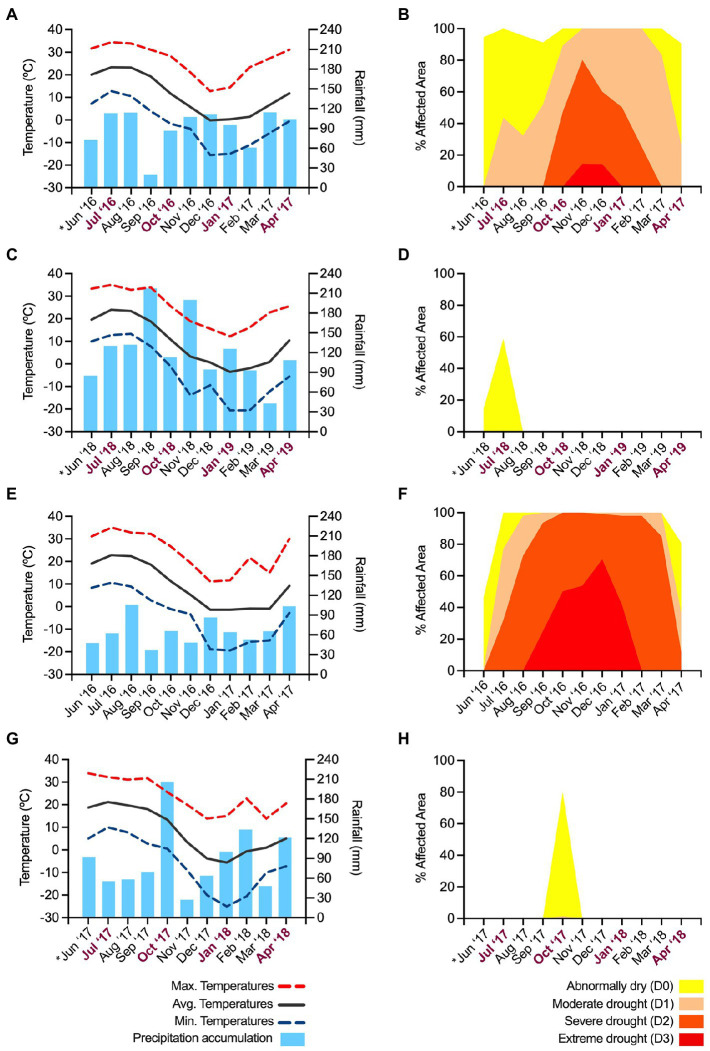
Summary of the environmental conditions during the assays. The first experiment repeat was performed in Highland, NY, during 2016–2017 **(A,B)**. The second experiment repeat with apple trees was carried out in the same orchards **(C,D)** in 2018–2019. The second repeat of the assays with pear and Asian pears was performed in (Belchertown, MA), in 2017–2018 **(G,H)**. The environmental conditions in Belchertown in 2016–2017 are also provided as a reference to compare conditions between the two locations during similar periods **(E,F)**. Charts on the left illustrate a monthly estimation of the maximum, average and minimum temperatures and the accumulated rainfalls during the 10-month assays. Data for graphs were retrieved from the SC-ACIS web service (https://scacis.rcc-acis.org). Because of the lack of complete data from the meteorological station in Highland throughout the two experiment repeats (2016–2019), the reported data are from the closest town (6.6 Km away) with fully available data, Poughkeepsie, NY, with similar elevation, latitude, and longitude coordinates as Highland. Charts on the right show an average monthly estimation of the intensity of the drought conditions and percent of the affected area in Ulster and Hampshire counties, where Highland **(B,D)** and Belchertown are located **(F,H)**. Drought graphs were created using data downloaded from the US drought monitor webpage (https://www.drought.gov). The *X*-axis of graphs A-D, G and H includes the timeline of the assays, and indicates the month in which tree inoculations were performed (*) and the points in which cankers were sampled (bold, purple letters).

The environmental conditions in Highland, NY, and Belchertown, MA, during the experiments are summarized in Figure 1. During the first experimental repeat with apple, pear and Asian pear trees in Highland, the weather was dominated by abnormally dry to extreme drought conditions. These conditions affected orchards in almost the entire Ulster County, NY, during the months in which the experiment was performed (from June 2016 until April 2017; Figures 1A,B). The reported drought was one of the most severe droughts on record, affecting the east coast of the U.S. and it also impacted Belchertown, MA (Figures 1E,F) in the same period, although no experiments were performed at that location in 2016.

The environmental conditions during the second experiment repeat on apples (June 2018 – April 2019) in Highland, NY (Figures 1C,D) and on pears and Asian pears (June 2017 – April 2018) in Belchertown, MA (Figures 1G,H) were in the range of what is considered normal compared to the past years. The only exceptions were abnormally dry periods in June and August in Highland in 2018 (Figure 1D) and in September and November in Belchertown in 2017 (Figure 1G). Despite these short abnormally dry periods, the weather during the second experimental repeat (Figures 1C,G) was rainier and colder than during the first experiment repeat (Figure 1A), particularly in the periods potentially affecting the outcome of the assays. For example, both locations showed around 17–23% higher accumulated rainfall in June (Figures 1C,G), when the inoculations and shoot infection development took place. A similar trend was also observed during most of the following months when canker formation and maturation took place. In September, 1 month before collecting the second batch of canker samples, the accumulated precipitations in Highland, NY (2018; Figure 1A) and Belchertown, MA (2017; Figure 1G) during the second experiment were 99 and 72% higher than in the same month during the first experiment repeat in 2016 (Figure 1A), respectively. Abundant precipitation events during the second repeat compared to the first one were also detected in October, January, February, and April. The percent increase of the accumulated rainfall in these months ranged between 5 and 58%.

In comparison to the first experiment repeat (Figure 1A), the average monthly temperatures during the second repeat in Highland, NY (Figure 1B) were between 0.5°C and 3.8°C colder in 7 out of 11 months in which the experiment took place. Similarly, temperatures in Belchertown, MA, during the second experimental repeat were from 1.2°C to 6.8°C lower than in the first repeat (Figure 1A) in 9 out of the 11 months in which the experiment was performed.

### Shoot blight and canker development

Overall, the severity of fire blight symptoms on apple, pear and Asian pear was similar in the two experimental repeats. Comparisons between irrigated and non-irrigated apple trees during this period did not reveal differences in the percentages of successful infections after shoot inoculation or the percentage of developed cankers in relation to the inoculated shoots (data not shown).

When considering the characteristics of the collected cankers, more than 97% of the cankers produced after shoot inoculation had a combination of cracked (determinate) and smooth margins (indeterminate type). All the results shown below originate from cankers with mixed types of margins, which were analyzed indistinctly.

### Canker sampling season, host cultivar, environmental conditions during the experiment and tree irrigation affect *Erwinia amylovora* detection rates in cankers

**Figure 2:**
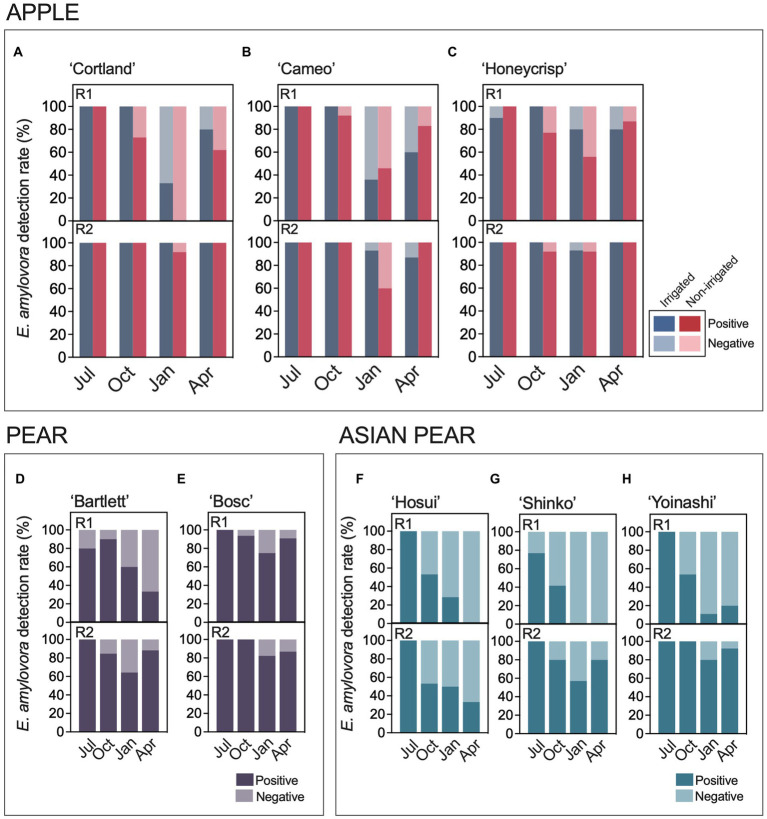
*E. amylovora* detection rates in cankers from irrigated and non-irrigated apple trees of cultivars ‘Cortland’ **(A)**, ‘Cameo’ **(B)**, and ‘Honeycrisp’ **(C)**, non-irrigated pear trees ‘Bartlett’ **(D)** and ‘Bosc’ **(E)**, and non-irrigated Asian pear trees ‘Hosui’ **(F)**, ‘Shinko’ **(G)** and ‘Yoinashi’ **(H)**. For each host species and cultivar, there are two graphs showing results from the experimental repeats R1, performed in 2016–2017 in Highland, NY; and R2, carried out in 2018–2019 in Highland, NY with apple, and in 2017–2018 in Belchertown, MA with pear and Asian pear. Sampling points: Jul, July; Oct, October; Jan, January; Apr, April.

Environmental and host-related factors might impact *E. amylovora* cell numbers in cankers, leading to variations in the v-dPCR detection rates. Figure 2 shows the percentages of positive and negative *E. amylovora* detections in cankers from different host species and cultivars. Around 100% of the cankers on apple (Figures 2A–C) harvested in July were positive for *E. amylovora* detections by v-dPCR, regardless of the apple cultivar, the experimental repeat, and the irrigation treatment (Figure 1). In cankers harvested in October and/or January, the ratio of positive detections was usually lower than in the previous months, especially in those coming from the first experiment repeat (R1, 2016–2017). In April, a general increase in the percent of positive cankers relative to January was observed in apple, ranging between 7 and 62%, regardless of the cultivar, irrigation treatment and experimental repeat. The trends observed during the second experimental repeat (R2, 2018–2019) were similar to the first experimental repeat (R1), but with higher percentages of positive cankers (Figure 1). Overall, there was a higher rate of cankers positive for *E. amylovora* detection on irrigated trees.

These results were supported by logistic regression analysis (Supplementary Table S3). A univariate approach confirmed separate effects of the apple cultivar, irrigation treatment, canker harvest season and the experimental repeat in *E. amylovora* detection by v-dPCR. The analysis by multivariable logistic regression showed that the model containing all the analyzed variables explained better the observed data than the models with one, two or three of the variables, with a positive and negative predictive power of about 89 and 77%, respectively (Supplementary Table S4).

All the Variance Inflation Factor (VIF) values associated with the variable levels in the final model were around 1, indicating that none of them contained redundant information (Supplementary Table S3). Compared to the reference levels of cultivar ‘Cortland’, non-irrigated trees, canker harvest in January, and the first experimental repeat, all the variable levels were positively associated with *E. amylovora* detection in cankers (Odds Ratio - OR-values above 0; Supplementary Table S3). This means that the studied predictors enhanced the chances of detecting *E. amylovora* in cankers compared to the selected reference levels. Primarily, the canker harvest period was the predictor with the highest effect on *E. amylovora* detection (higher OR values), followed by the experimental repeat, the host cultivar, and the irrigation treatment. For example, the model indicated that, after accounting for the other variables, the chances of detecting live *E. amylovora* cells in cankers collected in July, October and April were 63.4, 12.2 and 4.9 times higher than in January (*p* < 0.0001), respectively. The chances of detecting *E. amylovora* in cankers formed during the second experimental repeat were around 11.6 times higher than in the first experiment repeat (*p* < 0.0001; Supplementary Table S3). Similarly, detecting *E. amylovora* in ‘Honeycrisp’ cankers was 186% more likely (OR = 2.86) than in the reference cultivar ‘Cortland’ (*p* = 0.0023). Differences between ‘Cameo’ and the reference ‘Cortland’ were non-significant (*p* = 0.5865). Finally, after adjusting for the effects of the apple cultivar, the canker harvest period and the experimental repeat, the analysis also indicated that irrigation enhanced 75.7% the chances of detecting *E. amylovora* in cankers compared to the control treatment (non-irrigated trees; *p* = 0.0423; Supplementary Table S3).

The variation of positive and negative *E. amylovora* detections in pear (Figures 2D,E) and Asian pear (Figures 2F–H) from summer to the spring of the next season was similar to that in apple (Figures 2A–C), but with some differences. The positive detections of the pathogen ranged from 80–100% in July, decreased in October and January, and continued dropping in April in some of the cultivars. These trends were mainly observed during the first experimental repeat when the fraction of positive cankers in two out of three Asian pear cultivars reached values of 0 in January and April (Figures 2F,G). These observations were supported by logistic regression analysis (Supplementary Tables S5, S6). The univariate modeling of the effects of the host cultivar, canker harvest season, and the experimental repeat showed a significant impact of the three separate variables on the v-dPCR *E. amylovora* detection in cankers on pear (*p* ≤ 0.0417; Supplementary Table S5) and Asian pear (*p* ≤ 0.019; Supplementary Table S6). As with apple, the multivariable models that better explained the results on pear and Asian pear included the three predictors: host cultivar, canker harvest season and experiment repeat. The Asian pear model generally showed better classification power and goodness-of-fit parameters than the pear model (Supplementary Tables S7, S8). The VIF values revealed no collinearity between the analyzed predictors.

After accounting for the canker harvest season and the experimental repeat, the multivariable model on pear cankers indicated 70% fewer chances of detecting *E. amylovora* in ‘Bartlett’ than in ‘Bosc’ cankers (OR 0.3039, *p* = 0.0036; Supplementary Table S5). In Asian pear, the chances to detect *E. amylovora* in cankers in ‘Shinko’ and the reference cultivar ‘Hosui’ were similar (*p* = 0.6178). However, detecting the pathogen in ‘Yoinashi’ cankers was around 3.2 times more likely than in ‘Hosui’ (*p* = 0.0025; Supplementary Table S6). In both pear and Asian pear, after accounting for the effects of the cultivar and the experimental repeat, the chances of detecting the pathogen in July were around 11 (*p =* 0.0028) and 73 (*p* < 0.0001) times higher than in January (reference), respectively. Similarly, detecting the *E. amylovora* in pear and Asian pear cankers collected in October was about 5 (*p* = 0.0079) and 4 times (*p* = 0.001) higher than in January, respectively (Supplementary Tables S5, S6). However, no differences were detected between January (reference level) and April (*p* ≥ 0.5362; Supplementary Tables S5, S6), indicating that there was no statistical evidence for recovery of positive *E. amylovora* detections in April as observed on apple (Supplementary Table S3).

Finally, we analyzed the differences in *E. amylovora* detection in cankers at the host species level by grouping data from all the cultivars belonging to the same host species (Supplementary Table S9). Since the pear and Asian pear trees were not connected to irrigation system, only non-irrigated apple trees were included in the analysis. The univariate approach of the logistic regression analysis confirmed the results observed when analyzing the host species individually (Supplementary Tables S3, S5, S6), i.e., there were independent effects associated with the season in which cankers were sampled (*p* < 0.0001), the experimental repeat (*p* < 0.0001), and also the host species (*p* < 0.0001). The model containing these three variables explained the results better than the models containing just one or two variables (Supplementary Table S10). After accounting for the effects of the canker harvesting season and experiment repeat, the chances of detecting *E. amylovora* in apple and pear cankers were around 5 times higher than in Asian pear cankers (*p* < 0.0001). The analysis also indicated that, when examining the effects of the host species and the remaining variables together, recovering *E. amylovora* from cankers in April was 2 times more likely than in January.

### *Erwinia amylovora* populations in cankers vary with the host species and the year season

**Figure 3:**
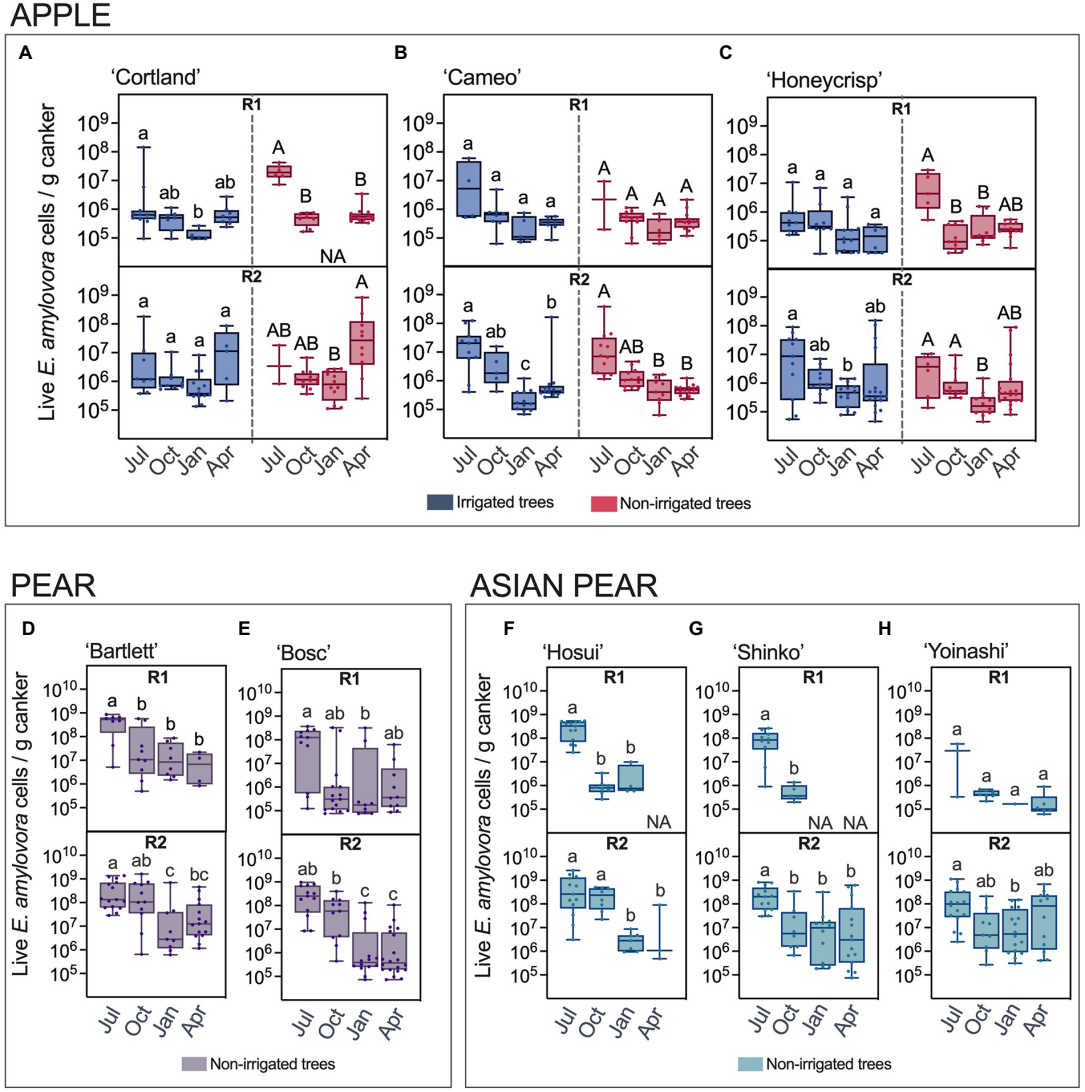
Live *E. amylovora* cell concentrations in cankers on irrigated and non-irrigated apple trees of the cultivars ‘Cortland’ **(A)**, ‘Cameo’ **(B)** and ‘Honeycrisp’ **(C)**, non-irrigated pear trees ‘Bartlett’ **(D)** and ‘Bosc’ **(E)**, and non-irrigated Asian pear trees ‘Hosui’ **(F)**, ‘Shinko’ **(G)** and ‘Yoinashi’ **(H)**. The median, first and third quartiles and the data range are indicated with box and whisker plots. Only cankers positive for *E. amylovora* detection were used in this analysis. Dots are *E. amylovora* concentrations in individual cankers. NA (not applicable) indicates that no positive detections were recorded in the specified cultivar/time point/experiment repeat. Different letters denote statistically significant differences (*p* < 0.05) between the *E. amylovora* concentrations in cankers collected in July (Jul), October (Oct), January (Jan), and April (Apr) assessed by Dunn’s multiple comparisons tests after Kruskal-Wallis ANOVA on ranks. In apple samples, lowercase and capital letters differentiate comparisons using data from irrigated and non-irrigated trees, respectively. Pairwise comparisons between the *E. amylovora* concentrations in cankers from irrigated and non-irrigated trees within each sampling point (Jul, Oct, Jan, Apr) were also included in the post-*hoc* analysis. These were not significant (α = 0.05) regardless of the experiment repeat or the apple cultivar. R1, first experiment repeat (2016–2017); R2, second experiment repeat (apple, 2018–2019, Highland, NY; pear and Asian pear, 2017–2018, Belchertown, MA).

The *E. amylovora* concentrations in apple cankers from irrigated and non-irrigated trees are shown in Figures 3A–C. The analysis of the results by a Kruskal-Wallis ANOVA test indicated the observed differences between the compared groups of samples were significant, regardless of the apple cultivar (*p* < 0.05). The pathogen populations in apple cankers in July were around 10^6^–10^7^ cells/g of canker. However, v-dPCR data showed a decline in *E. amylovora* populations of one to more than two orders of magnitude from July through January. Most of these observations were supported by posthoc Dunn’s tests that confirmed the significance of the bacterial population drop between July and January. These results were supported by posthoc Dunn’s multiple pairwise comparisons, which in most cases, confirmed the significance of the bacterial population drops between July and January. The visual analysis of the median *E. amylovora* populations indicated a partial recovery of the pathogen concentrations in April with respect to January, especially in ‘Cortland’ cankers from non-irrigated trees (Figure 3A). However, the posthoc analysis of the results indicated these differences as not significant in most cases.

The statistical analyses did not reveal significant differences in *E. amylovora* populations between irrigated and non-irrigated trees for any of the canker sampling time points, regardless of the cultivar, the experiment repeat, or the combination of data from R1 and R2 (*p* > 0.05).

*E. amylovora* populations in pear and Asian pear cankers over time are shown in Figures 3D,E and Figures 3F–H, respectively. The analysis of the data by Kruskal-Wallis tests revealed differences between the pathogen concentrations at the different sampling points, these differences being significant for each cultivar and experimental repeat (*p* ≤ 0.0272). In both *Pyrus* spp., we observed the highest pathogen cell densities in July, between 10^8^ and 10^9^ cells/g, and a decline of bacterial populations in two or more orders of magnitude during the following months. The recovery of the cell concentrations in April was not significant for any of the cultivars in the two *Pyrus* spp. (*p ≥* 0.8733). Instead, the general trend consisted of a progressive decline of the *E. amylovora* populations after July, even reaching undetectable values in April in two out of the three Asian pear cultivars in the first experimental repeat (R1; Figures 3F,G).

Data analysis also indicated that, in general, *E. amylovora* populations in the second experimental repeat (R2) were higher than in the first one (R1; Supplementary Tables S11–S13). This trend was consistent among samples, regardless of the *E. amylovora* host plant species and cultivar. Moreover, differences between the two experiment repeats were more evident in cankers collected from October to April. Additionally, in apple samples, the higher populations in R2 were more significant (lower *p* values) in non-irrigated than in irrigated trees, especially in the cultivar ‘Cortland’ (Supplementary Table S11).

**Figure 4:**
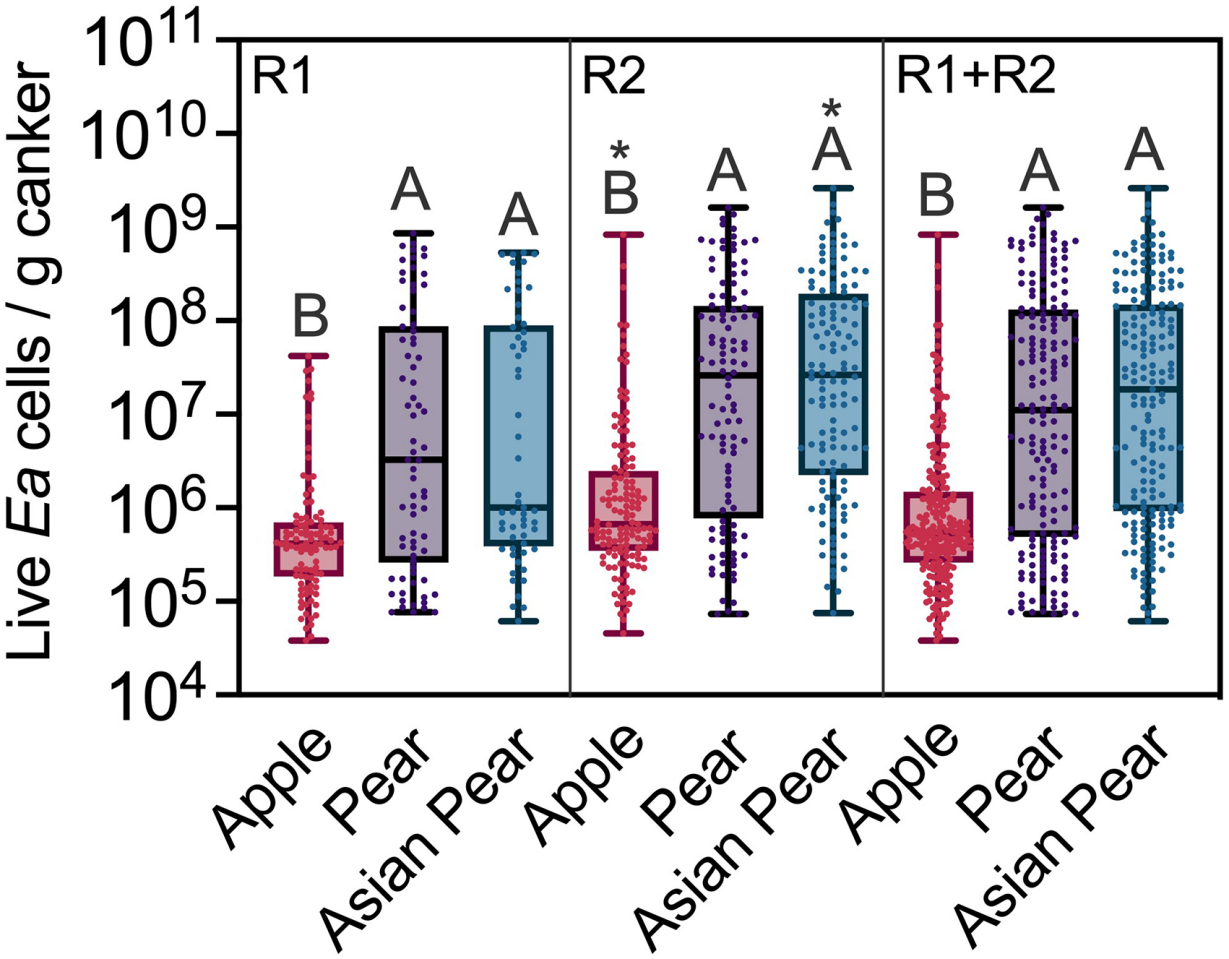
*Erwinia amylovora* live cell concentrations in cankers on apple, pear, and Asian pear, in an experiment repeated twice. Median values, interquartile ranges and full ranges of live *E. amylovora* cell concentration data are represented with box and whisker diagrams. Dots indicate concentration values for individual positive cankers for *E. amylovora* detection. The data sets labeled as apple, pear and Asian pear include data from all the cultivars belonging to the same host species. Different capital letters indicate significant differences between *E. amylovora* cell concentrations in apple, pear, and Asian pear cankers, assessed by Kruskal-Wallis ANOVA followed by Dunn’s multiple comparisons tests. All the trees used for these comparisons were non-irrigated. Asterisks denote significant differences between the datasets in the second experimental repeat (R2) and the same ones assessed during the first experimental repeat (R1). R1 + R2, combined data from R1 and R2.

Global comparisons between the *E. amylovora* populations in cankers of apple, pear and Asian pear are shown in Figure 4. For these comparisons, we only used data from non-irrigated trees, clustering together the data from the cultivars belonging to the same *E. amylovora* host species. Overall, the median *E. amylovora* populations in apple cankers were consistently lower and more compact than in the two *Pyrus* spp. This trend was more evident when pooling data from the two experimental repeats. The median *E. amylovora* concentrations in apple cankers were 5.35 × 10^5^ (IQR 2.60 × 10^5^–1.48 × 10^6^) live cells/g, in pear was 1.11 × 10^7^ (IQR, 4.88 × 10^5^–1.31 × 10^8^) live cells/g, and in Asian pear was 1.84 × 10^7^ (IQR, 9.25 × 10^5^–1.49 × 10^8^) live cells/g. Based on these numbers, only 25% of apple cankers contained more than 1.48 × 10^6^ live *E. amylovora* cells/g, while in pear and Asian pear, more than 50% of the cankers contained concentrations of the pathogen higher than that value. Differences between the median *E. amylovora* population values were smaller in R1 (of around 0.6 orders of magnitude) than in R2 (around 1.6 orders of magnitude) but highly significant in both experiment repeats (*p* < 0.0001). *E. amylovora* canker populations in pear and Asian pear were similar regardless of the year in which experiments were performed (*p* ≥ 0.7610). Similar to what we reported for individual cultivars, samples from the second experiment repeat (R2) contained higher *E. amylovora* populations than those from R1. However, differences between R1 and R2 were significant only for apple (*p* = 0.0263) and Asian pear (*p* = 0.0089), but not for pear (*p* > 0.0833; Figure 4).

In both experimental repeats, *E. amylovora* concentrations in apple cankers were also more concentrated around the median than in pear and Asian pear cankers (Figure 4). Using the IQR as a measure of data dispersion and the example of R1 + R2 data values from the paragraph above, 50% of apple canker data values from R1 + R2 varied 0.76 orders of magnitude around the median. In contrast, the *E. amylovora* concentration values in 50% of pear and Asian pear cankers varied 2.4 and 2.2 orders of magnitude around the median, respectively.

### Fire blight canker sizes vary with the host species and the environmental conditions during canker formation and development

**Figure 5:**
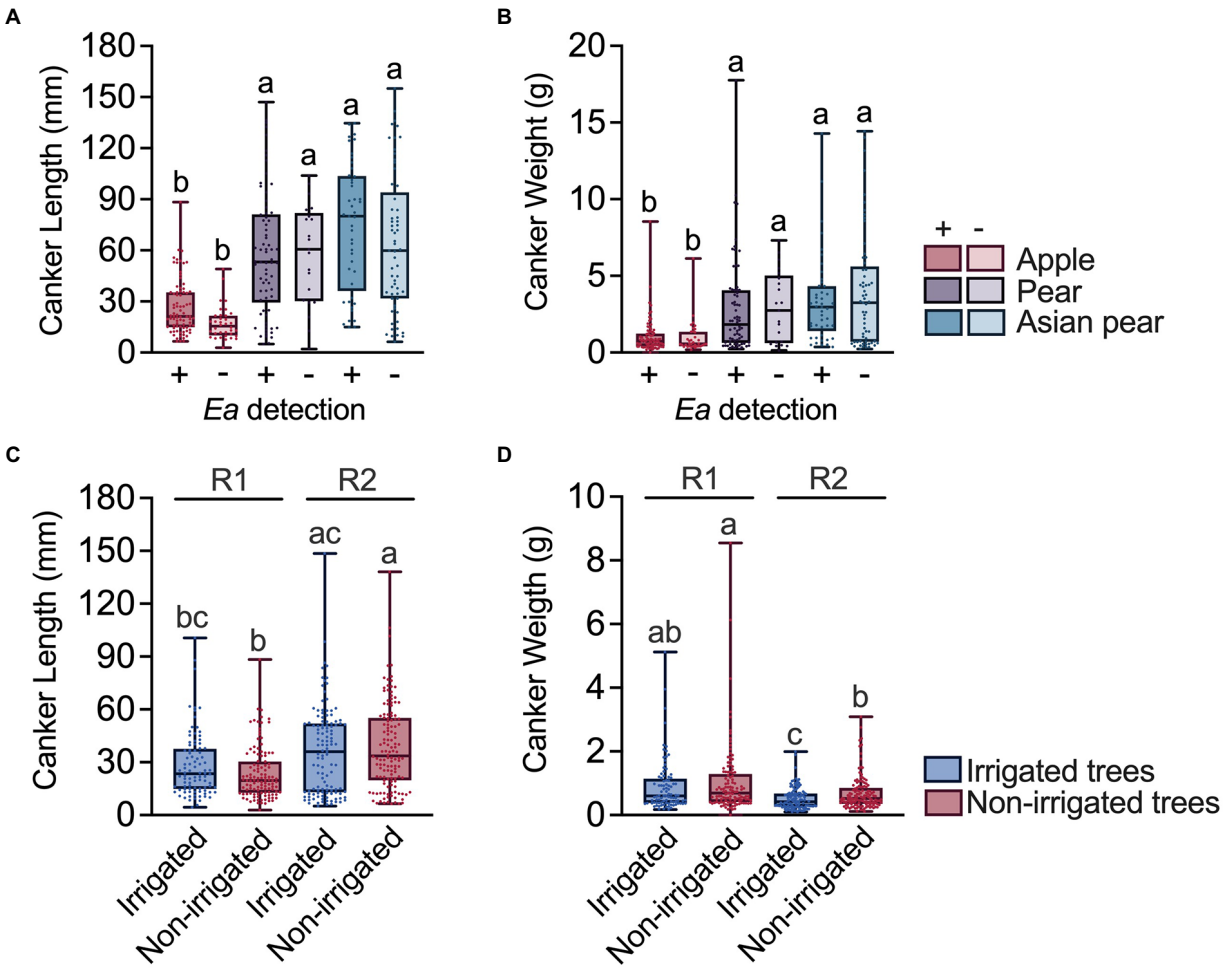
Relationship between the canker size and the *E. amylovora* host species, positive/negative detection of the pathogen in cankers, tree irrigation and the conditions during the experiment. Charts **(A)** and **(B)** show differences between the canker sizes in positive (+) and negative (−) samples for *E. amylovora* detection during the first experimental repeat, using apple, pear and Asian pear cankers from non-irrigated trees. Individual data for canker sizes are represented with dots. Boxes and whiskers illustrate the median, first and third quartiles and the range. Charts **(C)** and **(D)** compare canker lengths and weights from irrigated and non-irrigated apple trees from two independent experiments (R1, 2016–2017; R2, 2018–2019). In the four charts, each box contains data from cultivars representative of each species: Apple cvs. ‘Cortland’, ‘Cameo’ and ‘Honeycrisp’; pear cvs. ‘Bosc’ and ‘Bartlett’; Asian pear cvs. ‘Hosui’, ‘Shinko’, and ‘Yoinashi’. Different letters denote statistically significant differences based on Dunn’s multiple comparisons tests after a Kruskal-Wallis test.

To explore relationships between the canker characteristics and host factors, we compared the sizes of a subset of cankers from apple, pear and Asian pear trees. First, we determined potential canker size variations associated with *E. amylovora* detection in cankers. We did not detect significant differences in canker size between cankers which were positive and negative for *E. amylovora* detection regardless of the host species (*p* ≥ 0.2982; Figures 5A,B). With median values of around 19.7 (IQR 12.7–30.5) mm length and weight of 0.6 (IQR 0.41–1.3) g, cankers from apple were significantly shorter (*p* < 0.0001) and lighter (*p* ≤ 0.0142) than cankers from pear and Asian pear trees (Figures 5A,B). The median pear and Asian pear canker lengths and weights were 54.6 (IQR 30.7–80.9) mm and 2.0 (IQR 0.7–4.1) g, and 68.2 (IQR 31.9–100.1) mm and 3.1 (IQR 0.9–4.8) g, respectively. Pear and Asian pear canker sizes did not differ statistically (*p* ≥ 0.1589).

We also used apple canker data to determine the possible effects of irrigation and the environmental/experimental conditions on the canker size. As shown in Figure 5C, cankers from irrigated trees were slightly longer than cankers from non-irrigated trees (18.8% longer in R1 and 6.8% longer in R2). However, these differences were not statistically significant (*p* ≥ 0.634). Still, irrigated and non-irrigated tree cankers from the second experiment (R2) were 53.8 and 71.1% longer, respectively, than the cankers sampled in R1, with only differences between non-irrigated trees being significant (*p* < 0.0001). The observed effect of irrigation and environmental conditions during the second repeat of the experiment had an opposite effect on canker weight (Figures 5B,D). Cankers from irrigated trees in experiments R1 and R2 were 13.0 and 19.2% lighter, respectively, than the cankers from non-irrigated trees, with these differences being statistically significant only in R2 (*p =* 0.0234; Figure 5D). Similarly, cankers from irrigated and non-irrigated trees from R2 were also 30% (*p* < 0.0001) and 24.6% (*p* = 0.019) lighter, respectively, than the ones collected during the first experiment (R1).

**Figure 6:**
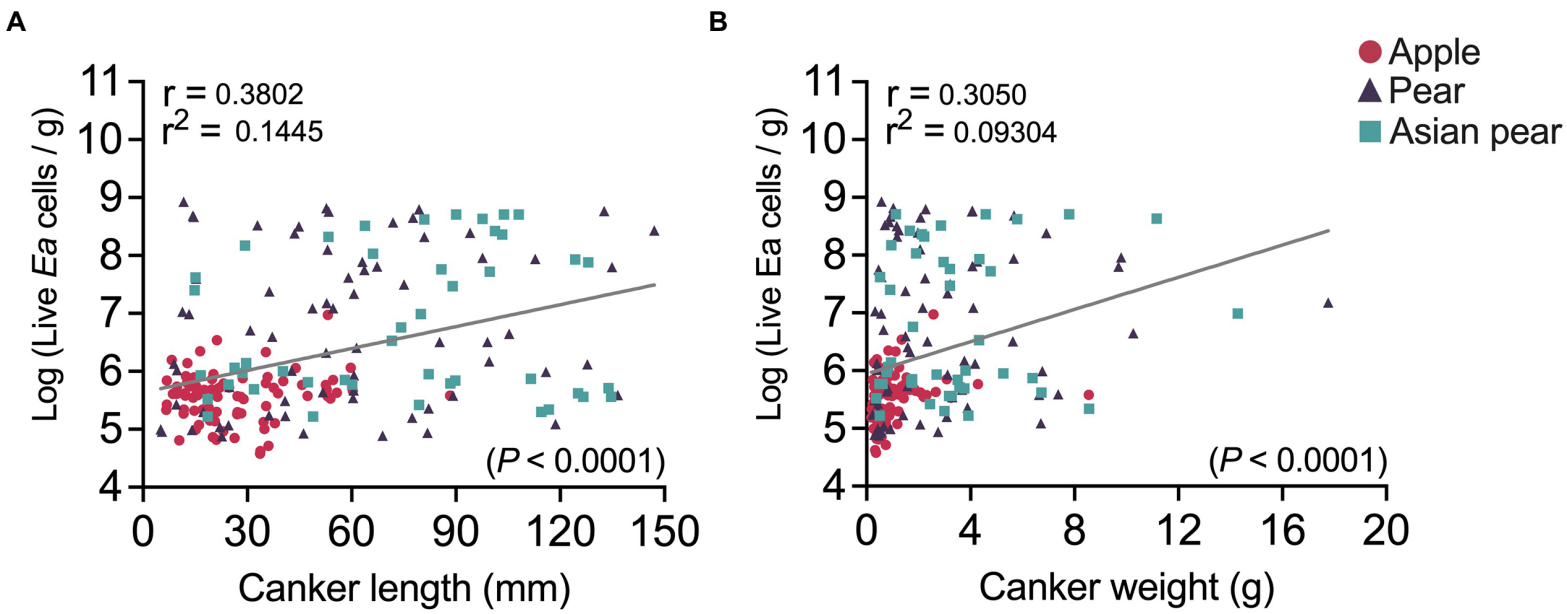
Correlation between fire blight canker sizes (length, chart **A**; weight, chart **B)** and *E. amylovora* live cell concentrations in cankers. Each chart shows the Pearson coefficient of correlation (*r*), coefficient of determination (*r*^2^), regression line and *p* value for the statistical testing. Circles, triangles, and squares indicate *E. amylovora* population sizes in individual cankers from apple (‘Cortland’, ‘Cameo’ and ‘Honeycrisp’), pear (‘Bosc’ and ‘Bartlett’), and Asian pear (‘Hosui’, ‘Shinko’, and ‘Yoinashi’), respectively. All the analyzed samples are from non-irrigated trees in the first experiment repeat. The negative cankers for *E. amylovora* detection were excluded from the analysis.

We also analyzed possible associations between the canker size and the *E. amylovora* cell concentrations in cankers. After pooling together canker data from apple, pear and Asian pear (non-irrigated trees in all cases), a correlation analysis showed a moderate-low but very significant (*p* < 0.0001) positive correlation between *E. amylovora* population sizes in cankers and the canker length (*r* = 0.38; Figure 6A) and weight (*r* = 0.31; Figure 6B). This indicates that longer and/or heavier cankers contained higher concentrations of the pathogen. Specifically, based on our results, 14.5 and 9.3% of the variability in the *E. amylovora* population sizes was explained by the variation of the canker length and weight, respectively. However, a similar analysis performed separately for each of the *E. amylovora* host species revealed very low and/or non-significant correlations between the same variables (data not shown). This result suggests that the effect of the canker size in *E. amylovora* concentrations is probably due to differences at the species and not the cultivar level. The age of the trees might have also affected the observed results, but more studies are required to evaluate this hypothesis.

### Positive *Erwinia amylovora* detections in pome fruit tree cankers correlate negatively with the host’s fire blight susceptibility

In most of our analyses, we observed differences in *E. amylovora* detection rates and population sizes linked to the host plant species and cultivar. To explore if these differences were related to varying levels of resistance or susceptibility to fire blight, we calculated a fire blight susceptibility index (FBSI) for each apple, pear and Asian pear cultivar used in our study. Then, we evaluated potential correlations between the FBSI, calculated using symptom severity and canker formation success data (incidence), and some of the variables analyzed in the previous sections.

Based on the FBSI, the Asian pear cultivars ‘Hosui’ and ‘Yoinashi’ were classified as extremely susceptible to fire blight (ES); the apple, pear and Asian pear cultivars ‘Cortland’, ‘Bartlett’, and ‘Shinko’, respectively, were classified as highly susceptible (HS); the apple and pear cultivars ‘Cameo’ and ‘Bosc’, respectively, were classified as susceptible (S); and the apple cultivar ‘Honeycrisp’, as moderately susceptible (MS). For a reference, we also used the FBSI to classify additional apple and Asian pear cultivars initially included in the first experimental repeat (Table 1). In general, the fire blight symptom severity on apple was low compared to pear and Asian pear, with some exceptions like the Asian pear cultivars ‘Olympic’ and, to some extent, ‘Shinko’.

**Table 1:** Fire blight resistance classification of the host species and cultivars used in this study.

**Host Species**	**Host Cultivar**	**NSI**^**a**^	**ECFI (% C/S)**^**b**^	**FBSI (NSI + ECFI)**^**c**^	**Classification**^**d**^
Apple	Cortland	3	4 (68.9%)	7	HS
	Cameo	3	2 (38.6%)	5	S
	Honeycrisp	2	2 (25.4%)	4	MS
	Red Delicious	1	1 (19.3%)	2	MR
Pear	Bosc	4	2 (28.5%)	6	S
	Bartlett	4	3 (57.0%)	7	HS
Asian Pear	Hosui	5	4 (74.0%)	9	ES
	Shinko	2	5 (96.4%)	7	HS
	Yoinashi	5	5 (91.8%)	10	ES
	Kosui	4	5 (83.2%)	9	ES
	Shinsui	4	2 (34.6%)	6	S
	Ya-Li	4	4 (69.5%)	8	HS
	Olympic	1	1 (8.0%)	2	MR

a NSI, Necrosis Severity Index, assigned after the visual comparison of fire blight symptoms (shoot blight onset, progression and spread to other parts of the tree) in non-irrigated trees of apple, pear and Asian pear cultivars inoculated in 2016. NSI ratings range from 0 to 5, where NSI = 0 means absence of fire blight symptoms; NSI = 1, very slow or no symptom progression after the onset of symptoms, and extremely rare death of branches in current year; NSI = 2, slow symptom progression and only occasional death of branches in current year; NSI = 3, faster symptom progression in comparison to cultivars classified as NSI = 2, with usual death of a small number of branches with cankers in current year; NSI = 4, faster symptom development in comparison to trees classified as NSI = 3, and frequent death of branches with cankers in current year; SSI = 5, the fastest symptom development in comparison to NSI = 1–4, and widespread death of branches or the whole tree in current year.

b ECFI, Effective Canker Formation Index. Ratings range from 0 to 5 and are defined by the percent of cankers formed relative to the number of blighted shoots (% C/S) recorded around 1 month after the inoculation. The percentages between parenthesis are average % C/S values from 5 to 6 tree replicates per cultivar. % C/S = 0, ECFI = 0; % C/S [1–20%], ECFI = 1; % C/S [21–40%], ECFI = 2; % C/S [41–60%], ECFI = 3; % C/S [61–80%], ECFI = 4; % C/S [81–100%], ECFI = 5.

c FBSI, Fire Blight Susceptibility Index, with values between 0 and 10, and calculated as NSI + ECFI.

d Based on the FBSI: FBSI = 0, resistant (R); FBSI [1, 2], moderately resistant (MR); FBSI [3, 4], moderately susceptible (MS); FBSI [5, 6], susceptible (S); FBSI [7, 8], highly susceptible (HS); FBSI [9, 10], extremely susceptible (ES).

Overall, the FBSI-based *E. amylovora* host classification, summarized in Table 1, was in line with previous studies (Supplementary Table S1). An exception was the Asian pear cultivar ‘Shinko’, which is usually reported as resistant or moderately resistant to fire blight (Supplementary Table S1), but in our analysis fell into the category of highly susceptible (HS) due to the high ratio of cankers developed relative to infected shoots (Table 1).

**Figure 7:**
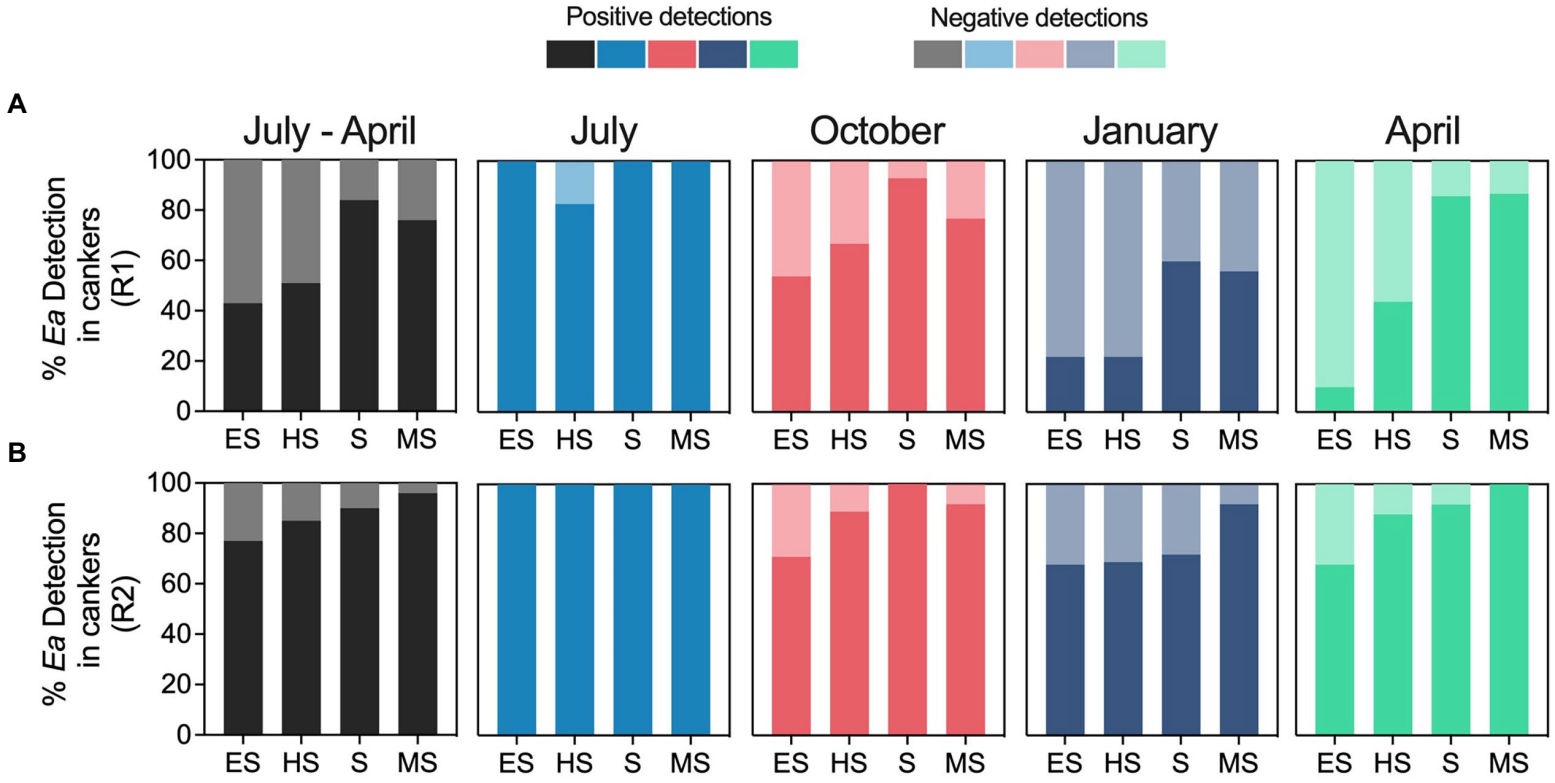
Relationship between the host’s susceptibility to fire blight and the *E. amylovora* detection rates in cankers over time. Charts show the percentages of positive and negative *E. amylovora* detection rates in cankers sampled from hosts classified as extremely susceptible (ES), highly susceptible (HS), susceptible (S) and moderately susceptible (MS) based on a fire blight susceptibility index (FBSI). Charts **A** and **B** show separated results of two independent experiments (R1 and R2, respectively). The black-colored charts on the left summarize E. amylovora detections rates in cankers pooling data from all the sampling points from July to April. The following four charts in each row, colored in pale blue, red, dark blue and green show separately the percentages of E. amylovora detections in cankers sampled in July, October, January, and April, respectively.

Figure 7 summarizes the relationship between the fire blight susceptibility of *E. amylovora* hosts and the pathogen detection rates in cankers. When analyzing the *E. amylovora* detection data from samples collected from July to April together, a trend of higher positive detection rates in cankers on the more resistant hosts was observed. This trend was evident in two independent experimental repeats (black bars in Figures 7A,B). The percent of positive detections in July was about 100% regardless of the host’s susceptibility to fire blight and the experimental repeat. In October, January, and especially April, the percentages of cankers positive for *E. amylovora* detection in the more resistant cultivars were up to 77% higher than in cultivars showing higher susceptibility to fire blight. We observed similar trends in the experiment repeats R1 and R2. These observations were supported by logistic regression analysis (Supplementary Table S14), which indicated that host resistance to fire blight, on its own, had a significant capacity to explain the obtained results. The univariate approach of the logistic regression analysis indicated that the chances of detecting *E. amylovora* in cankers from susceptible and moderately susceptible hosts were almost 4 times higher than in cankers from extremely susceptible hosts (*p* < 0.0001). The odds of detecting the pathogen in hosts classified as highly susceptible were statistically the same as in the extremely susceptible hosts (*p* = 0.1736). As reported in previous paragraphs, the variables “sampling season” and “experiment repeat,” analyzed individually, strongly affected the chances of detecting *E. amylovora*. When we incorporated these three variables (host resistance, canker harvesting season, and experiment repeat) into a multivariable model, the model showed better goodness-of-fit parameters (Supplementary Table S15). This indicates that fire blight resistance impacts but is not the only parameter affecting *E. amylovora* persistence in cankers. As described for the univariate model, the multivariable analysis indicated a positive relationship between the host resistance to fire blight and the pathogen detection rates. For example, after accounting for the effects of the canker harvesting season and the experiment repeat, the model showed that the chances of detecting *E. amylovora* in highly susceptible, susceptible, and moderately susceptible hosts were around 2, 6 and 7 times higher than in extremely susceptible hosts, respectively (*p* < 0.0001; Supplementary Table S14).

A separate analysis for each sampling season (Supplementary Table S16) confirmed the trends observed in Figure 7. In July, the analysis did not assign OR values to any of the variables because most of the cankers contained detectable levels of live *E. amylovora* cells regardless of the host resistance to fire blight or the experimental repeat. However, after July, the data showed the higher ORs associated with the more resistant cultivars to fire blight. For example, the logistic regression model for cankers collected in October revealed that, after accounting for the effects of the experimental repeat, the highest ORs for *E. amylovora* detection in cankers were associated with susceptible (OR 17.6; *p* = 0.0003) and moderately susceptible hosts (OR 3.5; *p* = 0.0448), in comparison to extremely susceptible hosts. *E. amylovora* detections in highly susceptible hosts were similar to those in extremely susceptible hosts (*p* = 0.0634). In winter (January), the highest chances of finding *E. amylovora* in cankers occurred in hosts classified as moderately susceptible (OR 4.2; *p* = 0.0079) and susceptible (OR 2.5; *p* = 0.0293), and this trend continued throughout Spring (April). In the last sampling point in April, the ORs calculated for the logistic model reached the highest values. The chances of detecting *E. amylovora* in cankers from highly susceptible, susceptible, and moderately susceptible hosts at this sampling point (after accounting for the effects of the experimental repeat) were around 4.3, 20.3 and 40.8 times higher, respectively, than from extremely susceptible hosts (*p* ≤ 0.0014). Interpreted together, these results suggest a positive selection for *E. amylovora* survival in cankers on the most resistant hosts, starting in October, and making it more likely to find the pathogen in these hosts in spring of the next growing season.

### Increased fire blight susceptibility of pome fruit trees is associated with higher *Erwinia amylovora* populations in cankers

**Figure 8:**
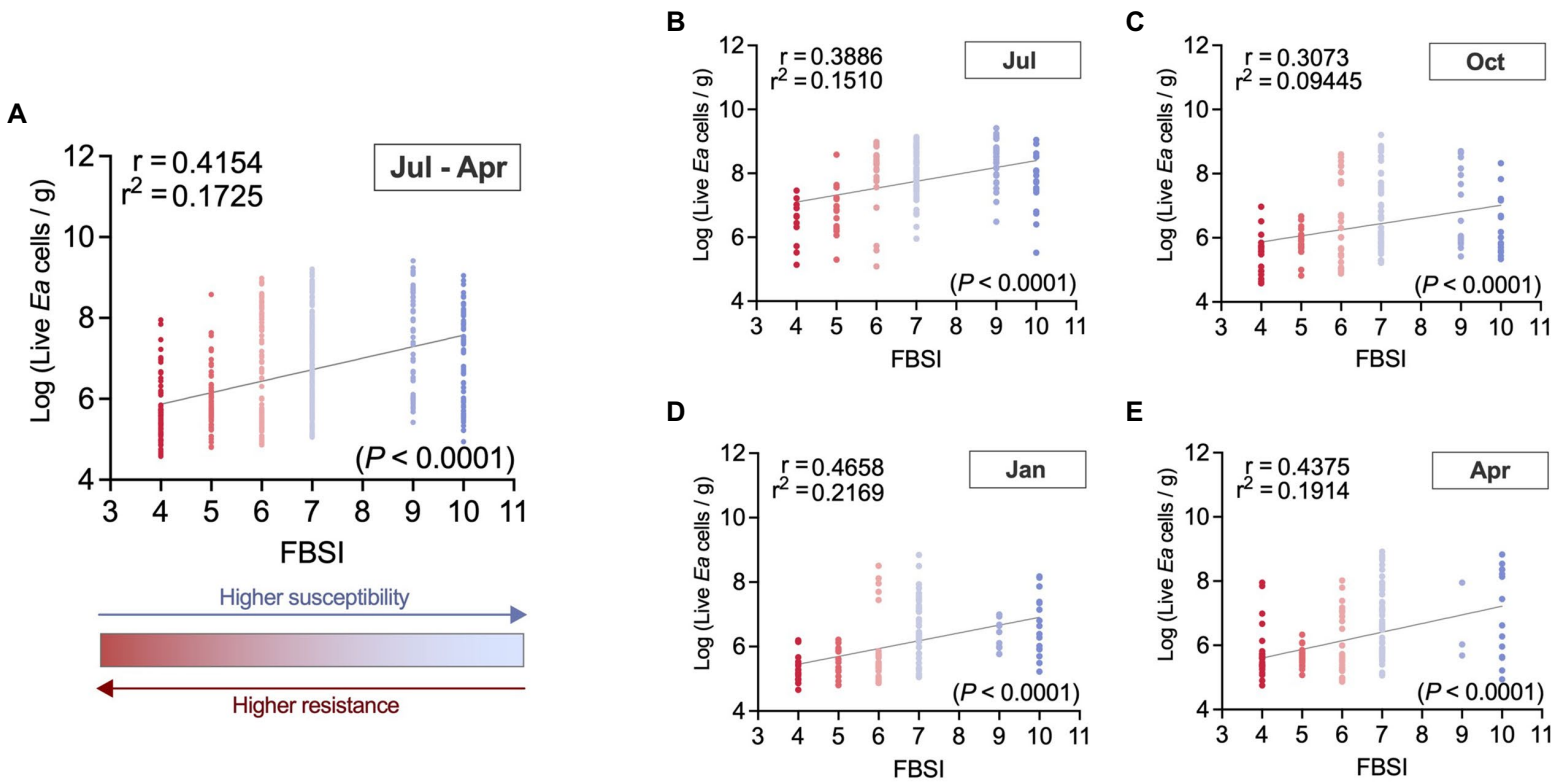
Correlation between the fire blight susceptibility index (FBSI) used in this study and *E. amylovora* population sizes in cankers collected in two independent experiments. Chart **(A)** describes the overall correlation using all samples collected in two independent experiments and all sampling season time points from July through April of the next year (*N* = 604). Charts **(B–E)** also contain pooled data from the two experiments but show the results from cankers collected in July (*N* = 153) **(B)**, October (*N* = 157) **(C)**, January (*N* = 129) **(D)** and April (*N* = 165) **(E)**. Dots correspond to live *E. amylovora* population data from individual cankers, colored to depict the host’s resistance/susceptibility to fire blight estimated with the fire blight susceptibility index (FBSI). Each chart shows Pearson’s coefficient of correlation (*r*), coefficient of determination (*r*^2^), regression line and *p* value.

Linked to the previous results, when we analyzed *E. amylovora* populations in cankers we found a moderate correlation between the host resistance to fire blight and the pathogen concentrations in canker tissues (Figure 8).

The correlation analysis of canker samples from two independent experiments collected from July through April of the next year (Figure 8A) revealed a positive (*r* = 0.42) and very significant (*p <* 0.0001) correlation between the live *E. amylovora* concentrations and the FBSI. In other words, we found higher *E. amylovora* population sizes associated with hosts showing higher susceptibility to fire blight. Based on the coefficient of determination, around 17.3% of the variance in the Log-*E. amylovora* concentrations were explained by variations of the FBSI. The highest and lowest correlations between the two variables were observed in samples from January (*r* = 0.47; Figure 8D) and October (*r* = 0.31; Figure 8C), with 21.7 and 9.4% of the variance in *E. amylovora* populations explained by the FBSI, respectively. These results were similar when data from each experiment repeat (R1, R2) were analyzed separately (Supplementary Figures S2, S3). The correlations between the FBSI and the pathogen concentrations in R2 (*r* = 0.43; *p* < 0.0001; Supplementary Figure S3A) were higher than in R1 (*r* = 0.36; *p* < 0.0001; Supplementary Figure S2A). During the first experiment repeat, the correlation between *E. amylovora* populations and the FBSI was only significant in samples collected in July (*r* = 39; *p* = 0.0026) and January (*r* = 0.37; *p* = 0.0214; Supplementary Figures S2B,D). In the second repeat of the experiment, the correlations were significant in each sampling time, with January and July being the months where the correlations reached the maximum (*r* = 0.49; *p* < 0.0001) and minimum values (*r* = 0.39; *p* < 0.0001), respectively (Supplementary Figures S2B,D).

### Genes *PR-1, PR-2, PR5,* and *PR-8* are induced in the bark in response to *Erwinia amylovora* infection

**Figure 9:**
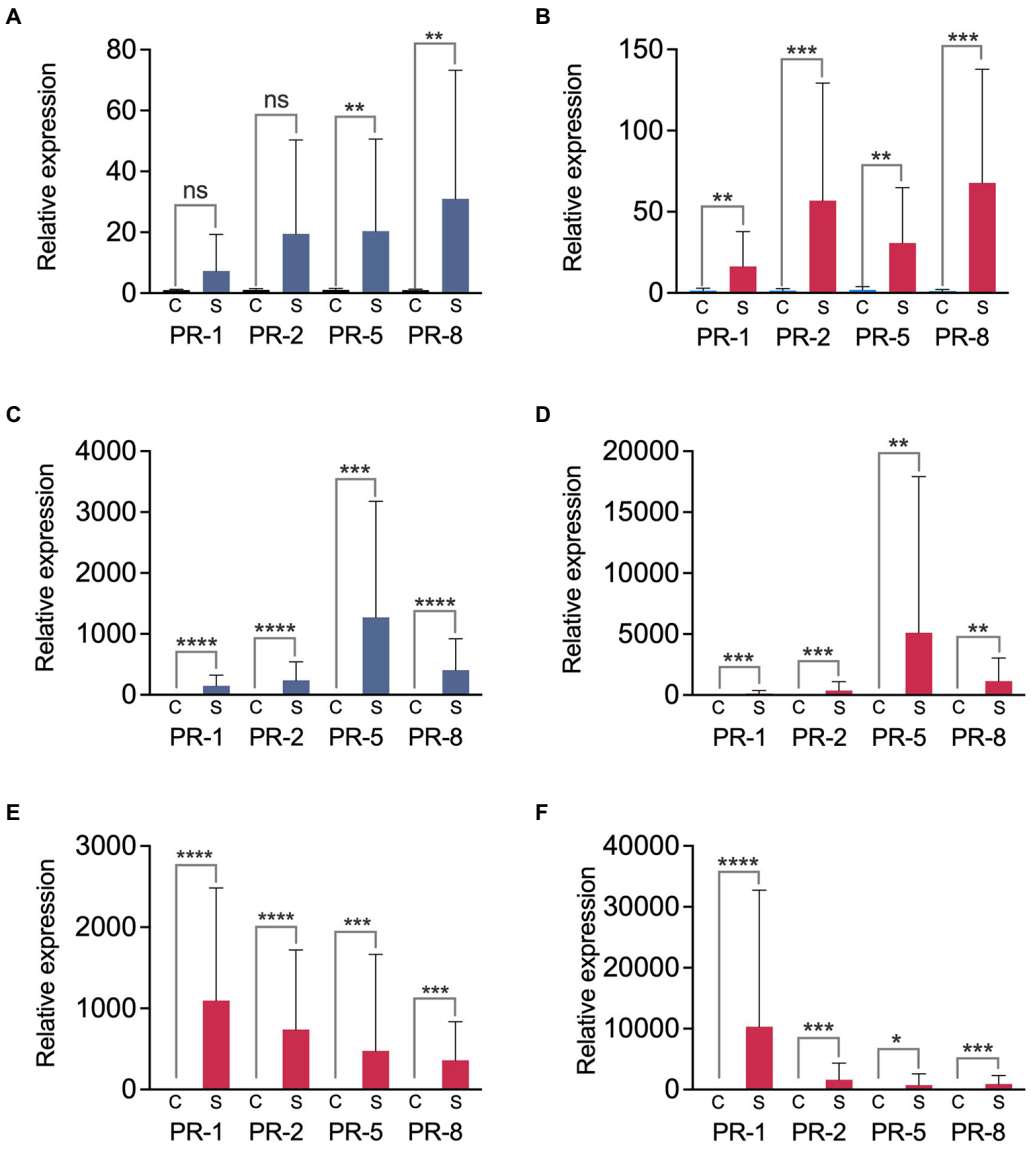
Relative expression of *PR* genes in the bark surrounding fire blight cankers. We analyzed bark samples from the fire blight highly susceptible and moderately susceptible apple cultivars ‘Cortland’ **(A,B)** and ‘Honeycrisp’ **(C,D)**, respectively, and from the Asian pear cultivars ‘Hosui’ (extremely susceptible) **(E)** and ‘Shinko’ (highly susceptible) **(F)**. Blue **(A,C)** and red columns **(B,D–F)** show the results obtained with cankers from irrigated and non-irrigated trees, respectively. Bars represent average relative expression values from two independent experiments, calculated by the 2^-ΔΔCt^ method, using actin gene as a calibrator. The error bars represent standard deviation (SD). Asterisks indicate significant differences between the relative expression of the indicated *PR* gene in the control (C) and in the bark samples near canker (S), assessed by two-tailed unpaired *t*-tests, *p < 0.05; **p < 0.01; ***p < 0.001; ****p < 0.0001; ns, not significant (*p* > 0.05).

The qPCR analysis revealed a general overexpression of the genes *PR-1, PR-2, PR-5* and *PR-8* in the bark surrounding apple and Asian pear fire blight cankers relative to the healthy plant tissue controls (*p* < 0.05; Figure 9). The high variability among samples made it difficult to assess differences between the relative expression values of the different genes. However, we observed two different expression patterns in the analyzed hosts.

In apple, the highest relative expression levels were observed in the genes *PR-5, PR-8,* and/or *PR-2* in comparison to *PR-1*, although these differences were not significant (one-way ANOVA, *p* ≥ 0.2835; Figures 9A–D). The moderately susceptible cultivar ‘Honeycrisp’ (Figure 9C,D) showed higher relative expression values for all the analyzed PR genes compared to the more susceptible cultivar ‘Cortland’ (Figure 9A,B; Supplementary Figures S4, S5). These differences were especially significant when comparing data from irrigated trees (unpaired *t-*tests, *p* ≤ 0.0166). Comparison between the *PR* gene relative expression values in cankers from irrigated and non-irrigated trees indicated no significant differences linked to the irrigation treatment, regardless of the apple cultivar or the experiment repeat (unpaired *t*-tests, *p ≥* 0.2729; Figure 9, Supplementary Figures S4, S5).

In Asian pear cankers, we also detected a significant induction of all the analyzed *PR* genes when compared to control samples (unpaired t-tests, *p* ≤ 0.0114; Figures 9E,F). The expression pattern was different than that observed in apple. In both Asian pear cultivars, *PR-5* and *PR-8* genes consistently showed the lowest relative expression levels, while *PR-1* was the most expressed gene. However, for an alpha of 0.05, the statistical analysis did not show significant differences between the expression values of the four genes in any of the cultivars (one-way ANOVA, *p ≥* 0.0699). Comparisons between the relative *PR* gene expression in the more susceptible and less susceptible Asian pear cultivars to fire blight did not reveal any clear relationship between the susceptibility to fire blight and the relative expression values. Depending on the experimental repeat and the analyzed PR gene, the extremely susceptible Asian pear cultivar ‘Hosui’ showed higher or lower relative expression values than the more resistant cultivar ‘Shinko’ (Figures 9E,F; Supplementary Figures S4, S5).

## Discussion

Since the first written reports of fire blight in the 18th century, cankers have been the subject of many studies focusing on their formation and development, the presence and dissemination of *E. amylovora*, and the role of holdover cankers in fire blight epiphytotics, etc. (Steiner, 1990; Schouten, 1993; Thomson, 2000; van der Zwet et al., 2012; Aćimović et al., 2014, 2021). The role of active cankers as inoculum sources for the epiphytotic spread of the pathogen is clear. However, cankers have also been found to serve as inoculum sources for latent infections on nearby shoots and buds *via* an endophytic route (van der Zwet, 1994; Hickey et al., 1999; Thomson, 2000). Some of the most obscure but critical segments of *E. amylovora* life cycle related to fire blight cankers, such as the pathogen survival, persistence, and interactions with various pome fruit hosts, as well as the effect of the environment on *E. amylovora* population dynamics in cankers, were not investigated for decades and are addressed in the current study.

Almost every apple, pear and Asian pear canker sampled in July contained *E. amylovora* cells in high concentrations. During the following months, the positive *E. amylovora* detection rates and live cell populations decreased progressively, usually reaching the lowest values in January. The drop in positive detections over time was probably due to a combination of factors. During the first stages of canker formation after shoot inoculation, the pathogen has access to nutrients in the healthy bark. The environmental temperatures at this time of the year are conducive to bacterial growth and infection, which leads to the necrosis of host plant cells. As *E. amylovora* cell numbers increase, while bacteria in the front line of the infection reach new susceptible plant tissues, the cells left behind get trapped within the collapsed plant cells. Under these conditions, *E. amylovora* is unable to grow or move to other tissues (Schouten, 1993). Nutrient scarcity, high temperatures over the summer, dryness and other stressing factors are probably related to the drop in *E. amylovora* cell numbers, as reported by previous works using controlled experimental conditions (Jock et al., 2005;Santander et al., 2014; Santander and Biosca, 2017). Exposure of the pathogen to cold and freezing temperatures during winter probably also contributed to the further decline in *E. amylovora* detections and live cell numbers, as demonstrated by assays performed by our group while determining the best conditions to preserve bacteria within cankers (Santander et al., 2022).

*E. amylovora* overwinters in fire blight cankers formed on branches diseased in the previous season. As temperatures rise in spring, the host renews growth, the pathogen multiplies in active canker margins, and bacterial cells emerge onto the bark surface (Gowda and Goodman, 1970; Schouten, 1993; Thomson, 2000; Zamski et al., 2006). In our study, we observed a significant increase of *E*. *amylovora* detections in April, at the beginning of the new growing season, which was more evident in fire blight cankers on apple. These data indicate that part of the cankers classified as negative for *E. amylovora* detection in January probably contained bacterial cells below the v-dPCR detection limit but remained active until the next growing season. Active cankers are usually identified by detecting *E. amylovora* cells or bacterial ooze on the canker surface. Although we did not visually examine if cankers were active in April, the observed increase of positive *E. amylovora* detections is in line with the pathogen multiplication in the host’s bark, preceding its release to the surface, reaching population sizes above the v-dPCR detection limit.

In our study, tree irrigation and probably rainier weather conditions during the second experimental repeat increased the chances of detecting *E. amylovora* in cankers. These results are congruent with those in other studies where the effects of moisture on *E. amylovora* recovery from cankers were evaluated. Beer and Norelli (1977) reported an improvement in the *E. amylovora* detection rates by covering cankers with wet pads weeks before attempting the isolation of the pathogen. The enhanced survival of the cells in a moist environment lacking nutrients compared to a dry environment has also been demonstrated (Jock et al., 2005). We also observed small but significant differences in the apple canker lengths and weights linked with the water availability to trees. The cankers developed during the first repeat of the experiment, under environmental conditions dominated by drought, were shorter and heavier than the cankers from the second experiment repeat. This effect was especially pronounced on cankers from non-irrigated trees. These results might be linked to the mechanisms by which the pathogen moves within the cortical parenchyma. The ooze produced by *E. amylovora* cells absorbs water from the apoplast. The osmotic pressure increase in the intercellular spaces causes water to leak from more plant cells and pushes *E. amylovora* bacteria in all directions throughout the neighboring parenchymatous tissues (Schouten, 1993; Zamski et al., 2006). We hypothesize that drought (and, in general, environmental dryness) might lead to enhanced water evaporation through the necrotic and cracked canker tissues. The deeper tissues in the bark, protected by the upper layers of dead plant cells, are thus probably the best sources of nutrients for *E. amylovora* to multiply and of water for the bacteria to move through the host tissues. Consequently, infections under these conditions probably tend to reach deeper layers of the bark, while evaporation restrains or makes it difficult for cankers to grow longitudinally.

The environmental temperatures probably also played a role in shaping the results during our field experiments. Beer and Norelli (1977) described lower active canker rates on trees incubated at 28°C than at 21°C. This agrees with the lower live *E. amylovora* cell detections and concentrations observed in cankers during the first experiment repeat, which was characterized by higher average temperatures than the second experiment repeat. The effect of temperature on the *E. amylovora* physiology depends on nutrient availability. The same temperatures optimal for growth and causing symptoms in host tissues are suboptimal for survival in nutrient-depleted environments. Similarly, suboptimal growth temperatures (e.g., 14°C) are optimal for pathogen survival under nutrient-limiting conditions (Santander et al., 2014; Santander and Biosca, 2017). During canker formation and development, while healthy plant tissues are still available, the warm temperatures in spring and summer are conducive to bacterial growth in the bark, leading to the onset and expansion of fire blight symptoms. However, *E. amylovora* cells trapped within necrosed tissues likely face nutrient scarcity, which is the most probable reason they do not multiply (Schouten, 1993; Sobiczewski et al., 2017). Under these conditions, the same temperatures enabling the pathogen to infect healthy plant cells, multiply, and move to other tissues, are detrimental to the long-term survival of *E. amylovora* cells trapped within the dead and nutrient-depleted tissues.

Previous works have shown differences among hosts in the number of cankers, proportion of active/inactive and determinate/indeterminate cankers produced (Beer and Norelli, 1977; Beer, 1978; Bonn, 1981; Biggs, 1994). In our study, mostly involving cankers with mixed determinate and indeterminate margins, we also found differences in canker sizes linked to the host species. Apple cankers were significantly smaller than the cankers formed on pears and Asian pears within the same period. We also found differences in the *E. amylovora* detection rates and/or viable cell concentrations in cankers associated with the host species and cultivar, and its susceptibility to fire blight. In this regard, the apple trees used in this study were around 10 years older than pear and Asian pear trees. The tree age is an important factor affecting the severity of fire blight disease symptoms (Smith, 2014). However, although this variable probably contributed to the observed results, we consider that the host species, environmental conditions, and canker sampling period were the variables having the strongest impact on our results, as deducted from the following facts: (i) despite the tree age, all the inoculations were performed in newly formed shoots; (ii) the higher *E. amylovora* detections and cell concentrations in the second experimental repeat or the lowest detection rates observed in January occurred similarly on apple, pear and Asian pear, regardless of the analyzed host species and tree age; *iii)* the host classification obtained with the FBSI mostly coincided with the previous classifications for different host species and cultivars within the same species; *iv)* cultivars of different host species fell into the same categories regardless of the tree age (e.g., ‘Cortland’, ‘Bartlett’ and ‘Shinko’ were classified as highly susceptible; ‘Cameo’, ‘Bosc’ and ‘Shinshui’ were recorded as susceptible; and ‘Red Delicious’ and ‘Olympic’ fell into the category of moderately resistant).

While our results revealed higher chances of detecting *E. amylovora* in cankers from more resistant than more susceptible hosts, further analysis showed a moderate positive correlation between the host susceptibility to fire blight and the live *E. amylovora* cell concentrations in cankers. The smaller *E. amylovora* population sizes in more resistant hosts were also reported by Milčevičová et al. (2010). The positive correlation between the host’s disease susceptibility and the pathogen populations is the general prediction in plant ecology studies, and is used to define the concepts of disease resistance (reduction of the pathogen growth, which translates into less severe symptoms) and disease tolerance (minor disease damage despite substantial pathogen populations) in plant-pathogen interactions (Schneider and Ayres, 2008; Pagán and García-Arenal, 2018).

The higher *E. amylovora* populations observed in cankers on more susceptible hosts seem counterintuitive to the lower *E. amylovora* detection rates in cankers observed in the same hosts. It is plausible that the higher pathogen concentrations and faster symptom development in susceptible hosts lead to higher pathogen populations trapped within dead plant tissues. As discussed above, once the pathogen cells are trapped within the necrosed bark tissues, exposure of starved *E. amylovora* cells to harsh environmental conditions such as dryness, heat, and cold temperatures from summer through winter quickly might decimate the pathogen populations. As a result, more *E. amylovora* cells would die in susceptible host cankers, leading to lower positive pathogen detection rates throughout October, January, and April. In contrast, *E. amylovora* cells in cankers on more resistant hosts reach lower cell concentrations, the tissue damage is less severe and progresses more slowly. These hosts thus maintain the bark structure and function for prolonged periods, which likely helps protect *E. amylovora* cells against challenging environmental conditions. Besides, *E. amylovora* EPS production induced by sorbitol during tissue invasion probably does not happen or is not as intense under nutrient-limiting conditions in necrosed tissues. The EPS produced by cells infecting green tissues also might confer additional protection against low and high temperatures, as described for other bacteria during interactions with plants (Morcillo and Manzanera, 2021), and against dehydration (Jock et al., 2005). All these data, together, might explain the increase of the pathogen detections in apple cankers in April compared to the more susceptible hosts pear and Asian pear.

The hypothesis described above also explains the similar *E. amylovora* detection rates observed in all the hosts in July. After shoot inoculation at the end of June, *E. amylovora* cells reaching the woody tissues have plenty of healthy plant material to grow on and multiply while the canker is formed. Regardless of the host resistance to fire blight, *E. amylovora* cells in this short period reach population sizes easily detected by v-dPCR.

The host resistance to fire blight is achieved, in part, by the release of plant defence molecules to the apoplast. Plant interactions with biotrophic pathogens usually activate the salicylic acid (SA) pathway. SA-signature gene products like some PR proteins are synthesized and accumulated locally and systemically as part of the SAR response. In previous works, researchers demonstrated the induction of different *PR* genes in flowers, leaves and shoots of apple in response to both *E. amylovora* (Bonasera et al., 2006; Milčevičová et al., 2010; Johnson and Temple, 2016) and chemical SAR inducers (Maxson-Stein et al., 2002; Aćimović et al., 2015). In our study, we demonstrated the local induction of the genes *PR-1, PR-2, PR-5,* and *PR-8* in perennial bark tissues surrounding cankers of different apple and Asian pear cultivars.

The expression patterns of the four analyzed *PR* genes differed depending on the host species. On apple, the genes *PR-5, PR-8,* and *PR-2* consistently showed higher expression levels than *PR-1*, while on Asian pear, *PR-1* was the most expressed gene. The observed differences between *PR* genes were not significant due to high expression variability among samples, but our results on apple were consistent with those reported by Malnoy et al. (2007). These authors demonstrated that *MpNPR1* gene overexpression in apple conferred enhanced resistance against fire blight and other apple diseases while activating the expression of *PR-2, PR-5,* and *PR-8.* However, it was unclear if one or more *PR* genes in the apple transformant lines overexpressing *MpNPR1* contributed to the enhanced resistance, what was the role of each gene and/or the participation of other genes in this phenotype. The genes *PR-2, PR-5,* and *PR-8* have also been reported to have anti-freezing properties and have been linked to cold tolerance in other plant models (Janská et al., 2010). Hence, their higher expression in apple might also contribute to creating a more favorable environment for *E. amylovora* to overwinter in cankers than in Asian pear.

We also observed consistently higher induction of the *PR* genes in the apple cultivar ‘Honeycrisp’ compared to the more susceptible cultivar ‘Cortland’. Verberne et al. (2000) suggested that higher *PR* gene expression values in more resistant cultivars might be linked to a higher accumulation of SA, leading to improved resistance to plant diseases. However, this trend was not evident in the Asian pear cultivars tested. We did not find any correlation between the relative expression values and the *E. amylovora* concentrations detected in more resistant and more susceptible host canker samples. The high variability in our results makes it difficult to make definitive conclusions about this topic and more work is required to determine the causes of the differences observed. In the future studies, including more housekeeping gene controls and increasing the number of samples might help reduce data variability.

Overall, we provide novel information about the *E. amylovora* population dynamics in cankers, the factors affecting the pathogen survival and persistence in these tissues over time, and the molecular responses of perennial tissues to *E. amylovora* infections. Our results highlight the potential role of hosts showing higher but not complete resistance to fire blight as more effective reservoirs of the pathogen in the field by favoring *E. amylovora* survival in cankers over the winter. Given that the host resistance to fire blight also depends on the age of the infected plant organ, the chances for the fire blight pathogen to overwinter successfully might also increase in cankers on older branches compared to the younger ones. Our results will pave the way for improving fire blight management options and invent novel eradication practices.

## Data availability statement

The raw data supporting the conclusions of this article will be made available by the authors, without undue reservation. The data presented in the study are deposited in the Figshare repository, accession https://doi.org/10.6084/m9.figshare.21202937.v1.

## Author contributions

RDS processed samples, designed, and prepared assays to validate dPCR data processed, and analyzed v-dPCR data, co-mentored ŽR, created graphs and tables, performed all the statistical analyses, drafted, reviewed and edited the manuscript. FK processed canker samples for v-dPCR samples, optimized qPCR and RNA extraction conditions, analyzed qPCR data, co-mentored ŽR and reviewed and edited the manuscript. CLM performed field inoculations, rated fire blight symptoms, collected data on symptom incidence in the field, collected and processed canker samples, and contributed to the experimental design of assays for dPCR validation. ŽR processed canker samples for qPCR, contributed to the optimization of qPCR and RNA extraction conditions, performed RNA extractions and qPCR assays, and contributed to dPCR sample processing. JC provided pear and Asian pear experiment orchard at the UMass Cold Spring Orchard in Belchertown, MA for the second experimental repeat, and reviewed the manuscript. SGA originated the project ideas and questions, wrote project proposals, and secured funding, designed the field experiments, tested and developed inoculation assays, performed field inoculations, collected cankers, provided the necessary laboratory equipment, supervised and coordinated the work, co-mentored ŽR and CLM, mentored RDS and FK, and reviewed and edited the manuscript. All authors contributed to the article and approved the submitted version.

## Funding

This material is based upon work supported by the National Institute of Food and Agriculture through New York State Specialty Crop Block Grant Program, project number SCG 82535/A001-SCG 17 005 to SGA in 2017, and by the National Institute of Food and Agriculture, U.S. Department of Agriculture, Hatch/Multi-State Project W4185, under Accession Number 1014444 Project Number NYG-625857, to SGA in 2017. This work was supported by the USDA National Institute of Food and Agriculture, Hatch Project Accession Number 1020561 to SGA, in 2019.

## Conflict of interest

The authors declare that the research was conducted in the absence of any commercial or financial relationships that could be construed as a potential conflict of interest.

## Publisher’s note

All claims expressed in this article are solely those of the authors and do not necessarily represent those of their affiliated organizations, or those of the publisher, the editors and the reviewers. Any product that may be evaluated in this article, or claim that may be made by its manufacturer, is not guaranteed or endorsed by the publisher.

## Footnotes

^1^ https://scacis.rcc-acis.org

^2^ https://www.drought.gov
