# Supplementary material for: Fire blight resistance, irrigation and conducive wet weather improve Erwinia amylovora winter survival in cankers
Source: Front Microbiol. 2022 Oct 18;13:1009364. doi: 10.3389/fmicb.2022.1009364 (PMC9623323; doi:10.3389/fmicb.2022.1009364)
Supplement: Supplementary file 1 [file Data_Sheet_1.docx]

Supplementary Material

# Supplementary Tables

**Supplementary Table S1.** *Erwinia amylovora* host plant species and cultivars included in this study. Blue bold letters show the apple, pear and Asian pear cultivars selected to assess *E. amylovora* survival in cankers in two independent experiment repeats. The remaining cultivars were removed from the main experiment but used to assess fire blight resistance in the different cultivars under our experimental conditions.

| **Host species** | **Apple (*Malus pumila* Mill.)** | **Pear (*Pyrus communis* L.)** | **Asian pear (*Pyrus pyrifolia* Nakai)** |
| --- | --- | --- | --- |
| **Host cultivar (resistance to Fire blight)^a^** | **‘Cortland' (HS - S)** | **‘Bartlett' (HS - MS)** | **‘Hosui’ (HS - MR)** |
|  | **‘Cameo’ (S)** | **‘Bosc' (HS - S)** | **‘Shinko’ (MR - HR)** |
|  | **‘Honeycrisp' (MR - HR)** |  | **‘Yoinashi’ (HS - MR)** |
|  | ‘Red Delicious (MS - R) |  | ‘Shinsui’ (MR - R) |
|  |  |  | ‘Ya-Li’ (S - R) |
|  |  |  | ‘Kosui’ (HS - R) |
|  |  |  | ‘Olympic’ (R, T) |

^a^ Ratings may vary depending on the growing/environmental/tree grafting conditions. Differences in fire blight resistance ratings reported by different sources, are represented as a range. The abbreviations used, in increasing order of resistance to fire blight, are HS, Highly Susceptible; S, Susceptible; MS, moderately Susceptible; MR, Moderately Resistant; Tolerant, T; R, Resistant; Highly Resistant, HR. The sources used to establish these categories are:

^-^ Bost, S., and Windham, A. (2002). SP277-R-Fire Blight, *The University of Tennessee Agricultural Extension Service*, SP277R-1.5M-7/02(Rev) E12-4615-00-012-03, <https://trace.tennessee.edu/utk_agexdise/34> (Accessed on March 2022)

^-^ Beckerman, J. (2006). BP-132-W Disease Susceptibility of Common Apple Cultivars. *Purdue University Extension Publication*, <https://mdc.itap.purdue.edu/item.asp?itemID=17721> (Accessed March 2022)

^-^ Beckerman, J. (2006). BP-30-W Disease Susceptibility of Common Apple Cultivars. *Purdue University Extension Publication*, <https://www.extension.purdue.edu/extmedia/BP/BP-30-W.pdf> (Accessed March 2022)

^-^ Crassweller, R., and Smith, D. (2012) <https://shaponline.org/wp-content/uploads/2012/03/Early-Results-of-an-Asian-Pear-Cultivar-Trial-Rob-Crassweller.pdf> (Accessed on March 2022).

^-^ Walsh, C.S., Harshman, J.M., Wallis, A.E., Williams, A.B., Newell, M.J. and Welsh, G.R. (G. R.. (2016). Asian pear: A potential alternative fruit crop for growers in the mid-Atlantic region. *HortScience*, 51, 1325–1328.

^-^ Skelly, J., and O’Callaghan, A. (2001) FS 01-56 Fire Blight. *University of Nevada, Reno, Extension.* <https://naes.agnt.unr.edu/PMS/Pubs/2001-3223.pdf> (Accessed March 2022).

^-^ Ivey, M. L. (2016) PLPATH-FRU-22 Fire blight of apples and pears, *Ohio State University Extension,* <https://ohioline.osu.edu/factsheet/plpath-fru-22-0> (Accessed March 2022).

^-^ Kushad et al., Apples and pears, In: R.T. Bessin, P.S. McManus, G.R. Brown, J.G. Strang (eds.), Midwest Tree Fruit Pest Management Handbook. (<http://www2.ca.uky.edu/agcomm/pubs/id/id93/ch_1.pdf>, Accessed on mARCH 2022).

^-^ Koski, R. D., and Jacobi, W. R. (2014) Fire blight, Fact sheet No. 2.907. *Colorado State University Extension.* <https://extension.colostate.edu/docs/pubs/garden/02907.pdf> (Accessed on March 2022).

**Supplementary Table S2.** List of primers used in this study.

| **Target gene** | **Primer ID** | **Primer Sequence (5' 🡪 3')** | **Source** |
| --- | --- | --- | --- |
| *PR-1* | aj708 | GTA GGC GTT GGT CCC TTG AC | Maxson-Stein et al (2002) |
|  | aj709 | GAT TGC AGT CGC CAA CAT GT |  |
| *PR-2* | aj778 | TCC GAT GCC ATT GCT TTT G | Maxson-Stein et al (2002) |
|  | aj779 | TTA TGG ACG AAA CGG CAA CA |  |
| *PR-5* | Forward | GGC AGG CGC AGT TCC ACC AG | Bonasera et al (2006) |
|  | Reverse | GAC ATG TCT CCG GCG TAT CA |  |
| *PR-8* | aj780 | CTC TTT TGA GCA GTT GGA ACC A | Maxson-Stein et al (2002) |
|  | aj781 | TGC CGG TAA CCC CAT GAA |  |
| *Actin* | aj748 | AAC TTC GTG TTG CTC CTG AAG AG | Maxson-Stein et al (2002) |
|  | aj749 | CAG TAG TAC GAC CAC TGG CAT AGA G |  |

**Table S3.** Associations between *E. amylovora* detection in apple cankers and the host cultivar, tree irrigation treatment, season in which samples were collected and the experiment repeat (N = 561).

|  |  | **Univariate logistic regression^a^** | | | |  |  | **Multivariable logistic regression^b^** | | |
| --- | --- | --- | --- | --- | --- | --- | --- | --- | --- | --- |
| **Variable and Levels** | | **Total^c^** | **Pos.^d^** | **Neg.^e^** | **OR (95% CI)** | ***P* value** |  | **OR (95% CI)** | ***P* value** | **VIF^f^** |
| Apple cultivar | |  |  |  |  |  |  |  |  |  |
|  | Cortland | 176 | 134 | 42 | Ref.^g^ |  |  | Ref. |  |  |
|  | Cameo | 185 | 150 | 35 | 1.343 (0.8113 to 2.235) | 0.2525 |  | 1.183 (0.6448 to 2.178) | 0.5865 | 1.387 |
|  | Honeycrisp | 200 | 179 | 21 | 2.672 (1.528 to 4.797) | **0.0007** |  | 2.861 (1.475 to 5.715) | **0.0023** | 1.39 |
| Irrigation treatment | |  |  |  |  |  |  |  |  |  |
|  | Irrigated trees | 265 | 228 | 37 | 1.6 (1.027 to 2.519) | **0.0395** |  | 1.757 (1.027 to 3.058) | **0.0423** | 1.027 |
|  | Non-irrigated trees | 296 | 235 | 61 | Ref. |  |  | Ref. |  |  |
| Canker harvesting period | |  |  |  |  |  |  |  |  |  |
|  | July (summer) | 90 | 89 | 1 | 49.71 (10.63 to 886.3) | **0.0001** |  | 63.4 (12.91 to 1148) | **<0.0001** | 1.288 |
|  | October (fall) | 123 | 115 | 8 | 8.029 (3.878 to 18.86) | **<0.0001** |  | 12.17 (5.471 to 30.41) | **<0.0001** | 1.345 |
|  | January (winter) | 173 | 111 | 62 | Ref. |  |  | Ref. |  |  |
|  | April (spring) | 175 | 148 | 27 | 3.062 (1.847 to 5.185) | **<0.0001** |  | 4.879 (2.701 to 9.109) | **<0.0001** | 1.393 |
| Experimental repeat | |  |  |  |  |  |  |  |  |  |
|  | R1 (2016-2017) | 283 | 198 | 85 | Ref. |  |  | Ref. |  |  |
|  | R2 (2018-2019) | 278 | 265 | 13 | 8.751 (4.908 to 16.85) | **<0.0001** |  | 11.56 (6.092 to 23.64) | **<0.0001** | 1.016 |

^a^ Determines if there is an association between *E. amylovora* detection and individual predictors. It does not take into consideration simultaneous effects of other predictor variables. The provided Odds Ratio and 95% Confidence Intervals, OR (95% CI), are thus unadjusted. The null hypothesis tested is that changes in the indicated variable would have no effects on the odds of *E. amylovora* detection. Significant *P* values (in bold letters) (α = 0.05) indicate an association between the analyzed factor and *E. amylovora* detection (outcome).

^b^ Analyzes the concurrent effect of the predictor variables and the outcome. As a result, the model provides adjusted OR (95% CI). Hence, *P* < 0.05 indicates a significant association between the variable level and the outcome, after adjusting for the effect of the other variables included in the model.

^c-e^ Total number of analyzed cankers in each group of samples, and number of cankers positive and negative for *E. amylovora* detection, respectively.

^f^ Variance Inflation Factor, used to estimate the potential collinearity between two or more of the variables included in the model. VIF values above 4 indicate high collinearity between variables in the model.

^g^ Reference level (baseline) for result interpretation.

**Supplementary Table S4.** Pairwise comparison between logistic regression models explaining the associations between *E. amylovora* detection in apple cankers and different predictor variables. The more complex model containing all the predictor variables (in the table, model 16) was compared to simpler models missing one or more variables (models 1 to 15). The table shows parameters related to model comparison and goodness-of-fit diagnostics.

|  |  | **Model diagnostics*** | | | | | | | |
| --- | --- | --- | --- | --- | --- | --- | --- | --- | --- |
| **Model no.** | **Model description** | **AICc** | **AUC** | **NPP (%)** | **PPP (%)** | **Tjur's R^2^** | **McFadden R^2^** | **H-L test** | **LogLR (G^2^) test** |
| 1 | Outcome ~ I | 521.8 | 0.5 | 0 | 82.53 | 8.55E-15 | 1.356E-14 | - | - |
| 2 | Outcome ~ I+CV | 513.3 | 0.6039 | 0 | 82.53 | 0.02139 | 0.02404 | >0.9999 | 0.0019 |
| 3 | Outcome ~ I+IRR | 519.4 | 0.5574 | 0 | 82.53 | 0.007635 | 0.00833 | >0.9999 | 0.0375 |
| 4 | Outcome ~ I+CHP | 454.5 | 0.7491 | 0 | 82.53 | 0.1211 | 0.141 | >0.9999 | <0.0001 |
| 5 | Outcome ~ I+REP | 455 | 0.7199 | 0 | 82.53 | 0.1115 | 0.1324 | >0.9999 | <0.0001 |
| 6 | Outcome ~ I+CV+IRR | 511.4 | 0.6254 | 0 | 82.53 | 0.02877 | 0.03161 | 0.6954 | 0.0009 |
| 7 | Outcome ~ I+CV+CHP | 446 | 0.7809 | 0 | 82.53 | 0.1523 | 0.1654 | 0.2385 | <0.0001 |
| 8 | Outcome ~ I+CV+REP | 449.4 | 0.7612 | 0 | 82.53 | 0.1332 | 0.1508 | 0.5555 | <0.0001 |
| 9 | Outcome ~ I+IRR+CHP | 451.6 | 0.7653 | 0 | 82.53 | 0.1327 | 0.1505 | 0.4965 | <0.0001 |
| 10 | Outcome ~ I+IRR+REP | 453.5 | 0.7442 | 0 | 82.53 | 0.1183 | 0.1391 | 0.9984 | <0.0001 |
| 11 | Outcome ~ I+CHP+REP | 383.4 | 0.8477 | 58.43 | 90.25 | 0.2728 | 0.2818 | >0.9999 | <0.0001 |
| 12 | Outcome ~ I+CV+IRR+CHP | 443.5 | 0.7909 | 58.62 | 84.77 | 0.1629 | 0.1741 | 0.1485 | <0.0001 |
| 13 | Outcome ~ I+CV+IRR+REP | 448.3 | 0.7721 | 0 | 82.53 | 0.1391 | 0.157 | 0.3257 | <0.0001 |
| 14 | Outcome ~ I+CV+CHP+REP | 376.4 | 0.8584 | 72.41 | 88.87 | 0.3035 | 0.3032 | 0.3462 | <0.0001 |
| 15 | Outcome ~ I+IRR+CHP+REP | 381 | 0.8594 | 58.43 | 90.25 | 0.2818 | 0.2904 | 0.3515 | <0.0001 |
| **16** | **Outcome ~ I+CV+IRR+CHP+REP** | **374.2** | **0.8679** | **72.41** | **88.87** | **0.3119** | **0.3113** | **0.1645** | **<0.0001** |

* Each column summarizes:

- Model number and model description. Outcome, positive *E. amylovora* detection; I, intercept; CV, host cultivar; IRR, irrigation treatment; CHP, canker harvesting period; REP, experiment repeat.

- AICc, Logistic regression model comparison (pairwise) by the corrected Akaike’s Information Criterion. The method determines how well the data support each model. The best models have the lowest AICc values. Models 1-15 were compared to the one containing all the variables (Model 16, in bold letters).

- AUC, Area Under the ROC curve. Evaluates the model’s classification performance (cutoff 0.5) in terms of sensitivity and specificity. Higher AUC values support a better classification performance of the model.

- NPP (%) and PPP (%), the model’s negative and positive prediction power, respectively. E.g., a PPP (%) = 60% indicates that 60% of the positives were correctly classified as positives by the model.

- Tjur’s R^2^ and McFadden’s R^2^ are pseudo R^2^ parameters used as indicators of the model’s goodness-of-fit (the higher their value, the better results are explained by the model). In general, the higher their value, the better fit. McFadden R^2^ between 0.2 and 0.4 represent an excellent fit ().

-HL, Hosmer-Lemeshow Test. Tests the null hypothesis that the specified model is correct. *P* values > 0.05 indicate that there is no evidence to reject the null hypothesis, i.e., the proposed model explains the results better than a model involving no effects associated with any of the variables).

- LRT, Log-likelihood Ratio test. Tests the null hypothesis that the intercept-only model fits the data best. *P* values < 0.05 indicate that there is evidence to think that the obtained data is better explained by the alternative model, i.e., the specified independent variables and interactions improve the fit of the model to the data.

**Supplementary Table S5.** Associations between *E. amylovora* detection in pear cankers by viability dPCR and the host cultivar, season in which samples were collected and the experimental repeat (N =220).

|  |  |  |  |  | **Univariate logistic regression^a^** | |  | **Multivariable logistic regression^b^** | | |
| --- | --- | --- | --- | --- | --- | --- | --- | --- | --- | --- |
| **Variable and Levels** | | **Total^c^** | **Pos.^d^** | **Neg.^e^** | **OR (95% CI)** | ***P* value** |  | **OR** | ***P* value** | **VIF^f^** |
| Pear cultivar | |  |  |  |  |  |  |  |  |  |
|  | Bosc | 114 | 103 | 11 | Ref.^g^ |  |  | Ref. |  |  |
|  | Bartlett | 106 | 80 | 26 | 0.3286 (0.1478 to 0.6888) | **0.0043** |  | 0.3039 (0.1316 to 0.6630) | **0.0036** | 1.002 |
| Canker harvesting period | |  |  |  |  |  |  |  |  |  |
|  | July (summer) | 49 | 47 | 2 | 9.744 (2.587 to 63.79) | **0.0034** |  | 10.62 (2.726 to 70.91) | **0.0028** | 1.435 |
|  | October (fall) | 50 | 46 | 4 | 4.768 (1.611 to 17.61) | **0.0087** |  | 5.101 (1.662 to 19.39) | **0.0079** | 1.442 |
|  | January (winter) | 58 | 41 | 17 | Ref. |  |  | Ref. |  |  |
|  | April (spring) | 63 | 49 | 14 | 1.451 (0.6403 to 3.334) | 0.3734 |  | 1.314 (0.5522 to 3.154) | 0.5362 | 1.499 |
| Experimental repeat | |  |  |  |  |  |  |  |  |  |
|  | R1 (2016-2017, Highland, NY) | 97 | 75 | 22 | Ref. |  |  | Ref. |  |  |
|  | R2 (2017-2018, Belchertown, MA) | 123 | 108 | 15 | 2.112 (1.036 to 4.409) | **0.0417** |  | 2.346 (1.089 to 5.188) | **0.0312** | 1.013 |

^a^ Determines associations between *E. amylovora* detection with separate predictors. The provided Odds Ratio and 95% Confidence Intervals, OR (95% CI), are unadjusted. Significant *P* values (in bold letters) (α = 0.05) indicate an association between the analyzed factor and the outcome.

^b^ Analyzes the effect of each predictor variable and the outcome after adjusting for the effect of the other variables included in the model. The model thus provides adjusted OR (95% CI). Bold letters (*P* < 0.05) show a significant association between the variable level and the outcome after accounting for the simultaneous effects of the other variables.

^c-e^ Total, positive and negative number of cankers for *E. amylovora* detection, respectively.

^f^ Variance Inflation Factor, used as an indicator of collinearity between the variables included in the model.

^g^ Reference level (baseline) for result interpretation.

**Supplementary Table S6.** Associations between *E. amylovora* detection in Asian pear cankers by viability dPCR and the host cultivar, season in which samples were collected and experimental repeat (N =302).

|  |  |  |  |  | **Univariate logistic regression^a^** | |  | **Multivariable logistic regression^b^** | | |
| --- | --- | --- | --- | --- | --- | --- | --- | --- | --- | --- |
| **Variable and Levels** | | **Total^c^** | **Pos.^d^** | **Neg.^d^** | **OR (95% CI)** | ***P* valuee** |  | **OR (95% CI)** | ***P* value** | **VIF^f^** |
| Asian pear cultivar | |  |  |  |  |  |  |  |  |  |
|  | Hosui | 106 | 58 | 48 | Ref.^g^ |  |  | Ref. |  |  |
|  | Shinko | 103 | 62 | 41 | 1.251 (0.7229 to 2.173) | 0.4236 |  | 1.197 (0.5894 to 2.440) | 0.6178 | 1.312 |
|  | Yoinashi | 93 | 66 | 27 | 2.023 (1.129 to 3.677) | **0.019** |  | 3.2 (1.525 to 6.925) | **0.0025** | 1.322 |
| Canker harvesting period | |  |  |  |  |  |  |  |  |  |
|  | July (summer) | 75 | 72 | 3 | 28.8 (9.756 to 123.8) | **<0.0001** |  | 72.88 (21.42 to 347.0) | **<0.0001** | 1.398 |
|  | October (fall) | 74 | 45 | 29 | 1.862 (0.9983 to 3.512) | 0.0522 |  | 3.704 (1.738 to 8.273) | **0.001** | 1.415 |
|  | January (winter) | 88 | 40 | 48 | Ref. |  |  | Ref. |  |  |
|  | April (spring) | 65 | 29 | 36 | 0.9667 (0.5060 to 1.842) | 0.9179 |  | 1.076 (0.5094 to 2.281) | 0.8472 | 1.367 |
| Experimental repeat | |  |  |  |  |  |  |  |  |  |
|  | R1 (2016-2017, Highland, NY) | 130 | 53 | 77 | Ref. |  |  | Ref. |  |  |
|  | R2 (2017-2018, Belchertown, MA) | 172 | 133 | 39 | 4.955 (3.027 to 8.237) | **<0.0001** |  | 9.231 (4.878 to 18.46) | **<0.0001** | 1.029 |

^a^ Determines associations between *E. amylovora* detection with separate predictors. The provided Odds Ratio and 95% Confidence Intervals, OR (95% CI), are unadjusted. Significant *P* values (in bold letters) (α = 0.05) indicate an association between the analyzed factor and the outcome.

^b^ Analyzes the concurrent effect of each predictor variable and the outcome, after adjusting for the effect of the other variables included in the model. The model provides adjusted OR (95% CI). Bold letters (*P* < 0.05) show a significant association between the variable level and the outcome after accounting for simultaneous effects of the other variables.

^c-e^ Total, positive and negative number of cankers for *E. amylovora* detection, respectively.

^f^ Variance Inflation Factor as an indicator of collinearity between the variables included in the model.

^g^ Reference level (baseline) for result interpretation.

**Supplementary Table S7.** Pairwise comparison between logistic regression models explaining the associations between *E. amylovora* detection in pear cankers and different predictor variables. The more complex model containing all the predictor variables (Model 8) was compared to simpler models missing one or more variables (Models 1 to 7). The table shows parameters related to model comparison and goodness-of-fit diagnostics.

|  |  | **Model diagnostics*** | | | | | | | |
| --- | --- | --- | --- | --- | --- | --- | --- | --- | --- |
| **Model no.** | **Model description** | **AICc** | **AUC** | **NPP (%)** | **PPP (%)** | **Tjur’s R^2^** | **McFadden R^2^** | **HL test** | **LRT** |
| 1 | Outcome ~ I | 201.3 | 0.5 | 0 | 83.18 | 4.44E-16 | -1.283E-15 | - | - |
| 2 | Outcome ~ I+CV | 194.5 | 0.6328 | 0 | 83.18 | 0.03951 | 0.04449 | >0.9999 | 0.0029 |
| 3 | Outcome ~ I+CHP | 189.7 | 0.6992 | 0 | 83.18 | 0.07385 | 0.08938 | >0.9999 | 0.0005 |
| 4 | Outcome ~ I+REP | 199.1 | 0.5924 | 0 | 83.18 | 0.01937 | 0.02125 | >0.9999 | 0.0396 |
| 5 | Outcome ~ I+CV+CHP | 182.1 | 0.7557 | 0 | 83.18 | 0.1211 | 0.1378 | 0.9979 | <0.0001 |
| 6 | Outcome ~ I+CV+REP | 192.2 | 0.6771 | 0 | 83.18 | 0.06538 | 0.06633 | 0.9305 | 0.0013 |
| 7 | Outcome ~ I+CHP+REP | 186.5 | 0.7407 | 0 | 83.18 | 0.1011 | 0.1157 | 0.8687 | 0.0001 |
| **8** | **Outcome ~ I+CV+CHP+REP** | **179.5** | **0.773** | **40** | **84.88** | **0.1502** | **0.1616** | **0.6539** | **<0.0001** |

* Each column summarizes:

- Model number and model description. Outcome, positive *E. amylovora* detection; I, intercept; CV, host cultivar; CHP, canker harvesting period; REP, experiment repeat.

- AICc, Logistic regression model comparison (pairwise) by the corrected Akaike’s Information Criterion. The method determines how well the data support each model. The best models have the lowest AICc values. Models 1-15 were compared to the one containing all the variables (Model 16, in bold letters).

- AUC, Area Under the ROC curve. Evaluates the model’s classification performance (cutoff 0.5) in terms of sensitivity and specificity. Higher AUC values support a better classification performance of the model.

- NPP (%) and PPP (%), the model’s negative and positive prediction power, respectively. E.g., a PPP (%) = 60% indicates that 60% of the positives were correctly classified as positives by the model.

- Tjur’s R^2^ and McFadden’s R^2^ are pseudo R^2^ parameters used as indicators of the model’s goodness-of-fit (the higher their value, the better results are explained by the model). In general, the higher their value, the better fit. McFadden R^2^ between 0.2 and 0.4 represent an excellent fit ().

-HL, Hosmer-Lemeshow Test. Tests the null hypothesis that the specified model is correct. *P* values > 0.05 indicate that there is no evidence to reject the null hypothesis, i.e., the proposed model explains the results better than a model involving no effects associated with any of the variables).

- LRT, Log-likelihood Ratio test. Tests the null hypothesis that the intercept-only model fits the data best. *P* values < 0.05 indicate that there is evidence to think that the obtained data is better explained by the alternative model, i.e., the specified independent variables and interactions improve the fit of the model to the da

**Supplementary Table S8.** Pairwise comparison between logistic regression models explaining the associations between *E. amylovora* detection in Asian pear cankers and different predictor variables. The more complex model containing all the predictor variables (Model 8) was compared to simpler models missing one or more variables (Models 1 to 7). The table shows parameters related to model comparison and goodness-of-fit diagnostics.

|  |  | **Model diagnostics** | | | | | | | |
| --- | --- | --- | --- | --- | --- | --- | --- | --- | --- |
| **Model no.** | **Model description** | **AICc** | **AUC** | **NPP (%)** | **PPP (%)** | **Tjur's R^2^** | **McFadden R^2^** | **HL test** | **LRT** |
| 1 | Outcome ~ I | 404.3 | 0.5 | 0 | 61.59 | 5.55E-16 | 2.967E-15 | - | - |
| 2 | Outcome ~ I+CV | 402.6 | 0.5749 | 0 | 61.59 | 0.01874 | 0.0143 | >0.9999 | 0.0563 |
| 3 | Outcome ~ I+CHP | 343 | 0.723 | 54.9 | 78.52 | 0.1826 | 0.1675 | >0.9999 | <0.0001 |
| 4 | Outcome ~ I+REP | 363.9 | 0.6894 | 59.23 | 77.33 | 0.1385 | 0.1053 | >0.9999 | <0.0001 |
| 5 | Outcome ~ I+CV+CHP | 336.5 | 0.7676 | 62.38 | 73.63 | 0.2182 | 0.194 | 0.4443 | <0.0001 |
| 6 | Outcome ~ I+CV+REP | 364.2 | 0.7233 | 57.89 | 70.53 | 0.1466 | 0.1149 | 0.1687 | <0.0001 |
| 7 | Outcome ~ I+CHP+REP | 291.6 | 0.8375 | 73.27 | 79.1 | 0.3458 | 0.3005 | 0.3304 | <0.0001 |
| **8** | **Outcome ~ I+CV+CHP+REP** | **284.8** | **0.8566** | **77.27** | **77.57** | **0.3738** | **0.3279** | **0.1343** | **<0.0001** |

* Each column summarizes:

- Model number and model description. Outcome, positive *E. amylovora* detection; I, intercept; CV, host cultivar; CHP, canker harvesting period; REP, experiment repeat.

- AICc, Logistic regression model comparison (pairwise) by the corrected Akaike’s Information Criterion. The method determines how well the data support each model. The best models have the lowest AICc values. Models 1-15 were compared to the one containing all the variables (Model 16, in bold letters).

- AUC, Area Under the ROC curve. Evaluates the model’s classification performance (cutoff 0.5) in terms of sensitivity and specificity. Higher AUC values support a better classification performance of the model.

- NPP (%) and PPP (%), the model’s negative and positive prediction power, respectively. E.g., a PPP (%) = 60% indicates that 60% of the positives were correctly classified as positives by the model.

- Tjur’s R^2^ and McFadden’s R^2^ are pseudo R^2^ parameters used as indicators of the model’s goodness-of-fit (the higher their value, the better results are explained by the model). In general, the higher their value, the better fit. McFadden R^2^ between 0.2 and 0.4 represent an excellent fit ().

-HL, Hosmer-Lemeshow Test. Tests the null hypothesis that the specified model is correct. *P* values > 0.05 indicate that there is no evidence to reject the null hypothesis, i.e., the proposed model explains the results better than a model involving no effects associated with any of the variables).

- LRT, Log-likelihood Ratio test. Tests the null hypothesis that the intercept-only model fits the data best. *P* values < 0.05 indicate that there is evidence to think that the obtained data is better explained by the alternative model, i.e., the specified independent variables and interactions improve the fit of the model to the data.

**Supplementary Table S9.** Associations between *E. amylovora* detection in pome fruit tree cankers (apple, pear, Asian pear) by viability dPCR and the host species, period of the year in which samples were collected and the experiment repeat (N =818).

|  |  |  |  |  | **Univariate logistic regression^a^** | |  | **Multivariable logistic regression^b^** | | |
| --- | --- | --- | --- | --- | --- | --- | --- | --- | --- | --- |
| **Variable and Levels** | | **Total^c^** | **Pos.^d^** | **Neg.^e^** | **OR (95% CI)** | ***P* value** |  | **OR (95% CI)** | ***P* value** | **VIF^f^** |
| Host species | |  |  |  |  |  |  |  |  |  |
|  | Apple | 296 | 235 | 61 | 2.413 (1.681 to 3.490) | **<0.0001** |  | 5.165 (3.320 to 8.175) | **<0.0001** | 1.307 |
|  | Pear | 220 | 183 | 37 | 3.085 (2.038 to 4.754) | **<0.0001** |  | 4.851 (2.972 to 8.095) | **<0.0001** | 1.269 |
|  | Asian pear | 302 | 186 | 116 | Ref.^g^ |  |  | Ref. |  |  |
| Canker harvesting period | |  |  |  |  |  |  |  |  |  |
|  | July | 158 | 153 | 5 | 24.43 (10.65 to 70.77) | **<0.0001** |  | 46.59 (19.08 to 141.0) | **<0.0001** | 1.374 |
|  | October | 198 | 157 | 41 | 3.077 (2.014 to 4.768) | **<0.0001** |  | 4.831 (2.959 to 8.055) | **<0.0001** | 1.41 |
|  | January | 232 | 129 | 103 | Ref. |  |  | Ref. |  |  |
|  | April | 230 | 165 | 65 | 2.027 (1.380 to 2.993) | **0.0003** |  | 2.175 (1.410 to 3.382) | **0.0005** | 1.441 |
| Experiment repeat | |  |  |  |  |  |  |  |  |  |
|  | R1 | 382 | 231 | 151 | Ref. |  |  | Ref. |  |  |
|  | R2^h^ | 436 | 373 | 63 | 3.854 (2.764 to 5.423) | **<0.0001** |  | 6.18 (4.143 to 9.396) | **<0.0001** | 1.013 |

^a^ Determines associations between *E. amylovora* detection with separate predictors. The provided Odds Ratio and 95% Confidence Intervals, OR (95% CI), are unadjusted. Significant *P* values (in bold letters) (α = 0.05) indicate an association between the analyzed factor and the outcome without considering other variables.

^b^ Analyzes the effect of each variable in the outcome while accounting for the simultaneous effect of the other variables included in the model. The provided OR (95% CI) values are thus adjusted values. Bold letters show a significant association (*P* < 0.05) between the variable level and the outcome while taking into consideration the concurrent effects of the other variables.

^c-e^ Total, positive and negative number of cankers for *E. amylovora* detection, respectively.

^f^ Variance Inflation Factor as a measurement of collinearity between the variables in the model.

^g^ Reference, baseline level for result interpretation.

^h^ We grouped together data from the second experiment repeat, regardless of the year and location where the inoculations were performed.

**Supplementary Table S10.** Pairwise comparison between logistic regression models explaining the associations between *E. amylovora* detection in pome fruit tree cankers and different predictor variables. The more complex model containing all the predictor variables (Model 8) was compared to simpler models missing one or more variables (Models 1 to 7). The table shows parameters related to model comparison and goodness-of-fit diagnostics.

| **Model no.** | **Model description** | **AICc** | **AUC** | **NPP (%)** | **PPP (%)** | **Tjur's R^2^** | **McFadden R^2^** | **HL test** | **LRT** |
| --- | --- | --- | --- | --- | --- | --- | --- | --- | --- |
| 1 | Outcome ~ I | 942.9 | 0.5 | - | 73.87 | 1.78E-15 | -7.491E-15 | - | - |
| 2 | Outcome ~ I+SP | 909.3 | 0.6267 | - | 73.87 | 0.04675 | 0.04002 | >0.9999 | <0.0001 |
| 3 | Outcome ~ I+CHP | 847.4 | 0.704 | - | 73.87 | 0.1062 | 0.1079 | >0.9999 | <0.0001 |
| 4 | Outcome ~ I+REP | 877.9 | 0.6611 | - | 73.87 | 0.08046 | 0.07123 | >0.9999 | <0.0001 |
| 5 | Outcome ~ I+SP+CHP | 799.4 | 0.7733 | 54.55 | 77.29 | 0.1734 | 0.1632 | 0.0337 | <0.0001 |
| 6 | Outcome ~ I+SP+REP | 834.5 | 0.7253 | 59.23 | 80.12 | 0.1408 | 0.1216 | 0.6289 | <0.0001 |
| 7 | Outcome ~ I+CHP+REP | 776.6 | 0.7795 | 63.81 | 79.41 | 0.1984 | 0.1853 | 0.9597 | <0.0001 |
| **8** | **Outcome ~ I+SP+CHP+REP** | **711.3** | **0.8273** | **65.32** | **84.37** | **0.2827** | **0.259** | **0.1532** | **<0.0001** |

* Each column summarizes:

- Model number and model description. Outcome, positive *E. amylovora* detection; I, intercept; SP, host species; CHP, canker harvesting period; REP, experiment repeat. Models were built using data from non-irrigated trees.

- AICc, Logistic regression model comparison (pairwise) by the corrected Akaike’s Information Criterion. The method determines how well the data support each model. The best models have the lowest AICc values. Models 1-15 were compared to the one containing all the variables (Model 16, in bold letters).

- AUC, Area Under the ROC curve. Evaluates the model’s classification performance (cutoff 0.5) in terms of sensitivity and specificity. Higher AUC values support a better classification performance of the model.

- NPP (%) and PPP (%), the model’s negative and positive prediction power, respectively. E.g., a PPP (%) = 60% indicates that 60% of the positives were correctly classified as positives by the model.

- Tjur’s R^2^ and McFadden’s R^2^ are pseudo R^2^ parameters used as indicators of the model’s goodness-of-fit (the higher their value, the better results are explained by the model). In general, the higher their value, the better fit. McFadden R^2^ between 0.2 and 0.4 represent an excellent fit ().

-HL, Hosmer-Lemeshow Test. Tests the null hypothesis that the specified model is correct. *P* values > 0.05 indicate that there is no evidence to reject the null hypothesis, i.e., the proposed model explains the results better than a model involving no effects associated with any of the variables).

- LRT, Log-likelihood Ratio test. Tests the null hypothesis that the intercept-only model fits the data best. *P* values < 0.05 indicate that there is evidence to think that the obtained data is better explained by the alternative model, i.e., the specified independent variables and interactions improve the fit of the model to the data.

**Supplementary Table S11.** Comparison between *E. amylovora* populations in cankers sampled in two experiment repeats, R1 (2016-2017) and R2 (2018-2019). Differences between R1 and R2 were assessed separately for each cultivar, canker harvesting period and irrigation treatment by two-tailed Mann-Whitney *U* tests (α = 0.05).

|  |  |  | **R1 (2016 - 2017)** | | **R2 (2018 - 2019)** | |  |  |  |  |
| --- | --- | --- | --- | --- | --- | --- | --- | --- | --- | --- |
| **Irrigation Treatment** | **Cultivar** | **Canker Harvest. Period** | **Median (Q1-Q3) (Cells/g)** | **n** | **Median (Q1-Q3) (Cells/g)^a^** | **n** | **Comparison** | **Mann-Whitney *U* Statistic** | **Sum Ranks R2, R1** | ***P value*^b^** |
| Irrigated trees | Cortland | July | 6.36E5 (4.53E5 - 8.97E5) | 11 | 1.20E6 (5.70E5 - 1.00E7) | 8 | R1 vs R2 | 28 | 94, 96 | 0.2060 |
|  |  | October | 5.04E5 (1.74E5 - 6.75E5) | 7 | **7.14E5 (6.36E5 - 1.48E6)** | 9 | R1 vs R2 | 10 | 38, 98 | 0.0229 (*) |
|  |  | January | 1.04E5 (9.39E4 - 1.95E5) | 5 | **3.74E5 (3.04E5 - 1.24E6)** | 14 | R1 vs R2 | 2 | 17, 173 | 0.0007 (***) |
|  |  | April | 5.23E5 (3.34E5 - 9.25E5) | 8 | **1.13E7 (7.31E5 - 5.14E7)** | 5 | R1 vs R2 | 9 | 45, 46 | 0.1274 |
|  |  |  |  |  |  |  |  |  |  |  |
|  | Cameo | July | 5.25E6 (5.48E5 - 4.78E7) | 4 | **2.03E7 (5.84E6 - 3.70E7)** | 11 | R1 vs R2 | 16 | 26, 94 | 0.4894 |
|  |  | October | 6.62E5 (3.56E5 - 8.13E5) | 8 | **1.84E6 (8.22E5 - 1.02E7)** | 5 | R1 vs R2 | 8 | 44, 47 | 0.0932 |
|  |  | January | 1.10E5 (8.38E4 - 5.80E5) | 5 | **1.63E5 (9.56E4 - 4.03E5)** | 12 | R1 vs R2 | 29 | 44, 109 | 0.9593 |
|  |  | April | 3.51E5 (2.68E5 - 4.67E5) | 9 | **4.35E5 (3.60E5 - 6.65E5)** | 13 | R1 vs R2 | 30 | 75, 178 | 0.0605 |
|  |  |  |  |  |  |  |  |  |  |  |
|  | Honeycrisp | July | 4.21E5 (2.05E5 - 9.79E5) | 9 | **1.57E7 (4.63E5 - 6.12E7)** | 11 | R1 vs R2 | 24 | 69, 141 | 0.0562 |
|  |  | October | 3.13E5 (2.60E5 - 1.14E6) | 9 | **9.02E5 (6.32E5 - 3.13E6)** | 11 | R1 vs R2 | 24 | 69, 141 | 0.0541 |
|  |  | January | 1.12E5 (4.10E4 - 2.57E5) | 12 | **4.68E5 (1.36E5 - 8.48E5)** | 14 | R1 vs R2 | 46 | 124, 227 | 0.0513 |
|  |  | April | 1.43E5 (3.81E4 - 3.17E5) | 8 | **3.49E5 (2.31E5 - 4.83E6)** | 18 | R1 vs R2 | 28 | 64, 287 | 0.0133 |
|  |  |  |  |  |  |  |  |  |  |  |
| Non-Irrigated trees | Cortland | July | 1.91E7 (1.28E7 - 3.33E7) | 6 | 3.39E6 (8.33E5 - 1.81E7) | 3 | R1 vs R2 | 3 | 36, 9 | 0.1667 |
|  |  | October | 5.00E5 (2.63E5 - 7.22E5) | 8 | **1.14E6 (7.36E5 - 1.91E6)** | 12 | R1 vs R2 | 12 | 48, 162 | 0.0041 (**) |
|  |  | January | 0.00E0 (0.00E0 - 0.00E0)^b^ | 3 | **7.97E5 (2.14E5 - 2.31E6)** | 12 | R1 vs R2 | 0 | 6, 144 | 0.0044 (**) |
|  |  | April | 5.32E5 (4.17E5 - 6.92E5) | 16 | **2.67E7 (3.78E6 - 1.25E8)** | 10 | R1 vs R2 | 16 | 152, 199 | 0.0003 (***) |
|  |  |  |  |  |  |  |  |  |  |  |
|  | Cameo | July | 2.21E6 (1.99E5 - 9.42E6) | 3 | **7.06E6 (1.70E6 - 3.17E7)** | 12 | R1 vs R2 | 11 | 17, 103 | 0.3648 |
|  |  | October | 5.32E5 (3.68E5 - 7.59E5) | 12 | **1.08E6 (6.10E5 - 2.18E6)** | 12 | R1 vs R2 | 24 | 102, 198 | 0.0045 (**) |
|  |  | January | 1.53E5 (8.04E4 - 4.82E5) | 6 | **4.06E5 (1.99E5 - 1.10E6)** | 9 | R1 vs R2 | 15 | 36, 84 | 0.1810 |
|  |  | April | 3.68E5 (2.28E5 - 4.93E5) | 14 | **4.85E5 (3.43E5 - 5.95E5)** | 13 | R1 vs R2 | 62 | 167, 211 | 0.1694 |
|  |  |  |  |  |  |  |  |  |  |  |
|  | Honeycrisp | July | 4.36E6 (1.31E6 - 2.29E7) | 5 | **6.72E6 (5.14E5 - 1.57E7)** | 6 | R1 vs R2 | 14 | 31, 35 | 0.9307 |
|  |  | October | 9.23E4 (4.92E4 - 3.77E5) | 10 | **5.36E5 (3.98E5 - 1.11E6)** | 12 | R1 vs R2 | 9 | 64, 189 | 0.0003 (***) |
|  |  | January | 1.47E5 (1.16E5 - 7.89E5) | 9 | **1.58E5 (9.32E4 - 3.24E5)** | 12 | R1 vs R2 | 49 | 104, 127 | 0.7544 |
|  |  | April | 2.50E5 (1.99E5 - 4.17E5) | 13 | **4.29E5 (2.40E5 - 1.20E6)** | 20 | R1 vs R2 | 82 | 173, 388 | 0.0784 |

^a^ Bold letters highlight the median cells/g in R2 that were higher than in R1, regardless of the result of the statistical analysis.

^b^ Asterisks indicate the level of significancy: *, *P* < 0.05; **, *P* < 0.01; ***, *P* < 0.001.

^c^ A 0% of the cankers collected from non-irrigated ‘Cortland’ trees were positive for *E. amylovora* detection. The absence of positives was arbitrarily substituted to perform the analysis by three replicates of 0 cell/g.

**Supplementary Table S12.** Comparison between *E. amylovora* populations in pear cankers sampled in two experiment repeats, R1 (2016-2017) and R2 (2017-2018). Differences between R1 and R2 were assessed separately for each cultivar and canker harvesting period by pairwise two-tailed Mann-Whitney *U* tests (α = 0.05).

|  |  | **R1 (2016 - 2017)** | |  | **R2 (2017 - 2018)** | |  |  |  |  |
| --- | --- | --- | --- | --- | --- | --- | --- | --- | --- | --- |
| **Canker Harvest. Period** | **Cultivar** | **Median (Q1-Q3) (Cells/g)** | **n** |  | **Median (Q1-Q3) (Cells/g)^a^** | **n** | **Comparison** | **Mann-Whitney *U* Statistic** | **Sum of Ranks (R1, R2)** | ***P value^b^*** |
| July | Bartlett | 5.30E8 (1.43E8 - 6.56E8) | 8 |  | 1.35E8 (6.07E7 - 7.08E8) | 15 | R1 vs R2 | 54 | 102 , 174 | 0.7284 |
|  | Bosc | 1.25E8 (5.36E5 - 2.43E8) | 11 |  | **2.52E8 (4.93E7 - 7.10E8)** | 13 |  | 43 | 109 , 191 | 0.1056 |
| October | Bartlett | 1.07E7 (2.65E6 - 2.67E8) | 9 |  | 1.06E8 (3.45E7 - 6.55E8) | 11 | R1 vs R2 | 25 | 70 , 140 | 0.0674 |
|  | Bosc | 3.08E5 (1.10E5 - 1.04E6) | 15 |  | **5.80E7 (4.52E6 - 1.71E8)** | 11 |  | 25 | 145 , 206 | 0.002 (**) |
| January | Bartlett | 8.41E6 (2.20E6 - 5.64E7) | 8 |  | 2.81E6 (1.15E6 - 4.06E7) | 9 | R1 vs R2 | 29 | 79 , 74 | 0.5414 |
|  | Bosc | 1.75E5 (8.08E4 - 4.54E7) | 9 |  | **3.93E5 (2.49E5 - 7.49E6)** | 14 |  | 39 | 84 , 192 | 0.1412 |
| April | Bartlett | 6.86E6 (9.67E5 - 1.95E7) | 4 |  | **1.26E7 (4.04E6 - 8.38E7)** | 15 | R1 vs R2 | 18 | 28 , 162 | 0.2621 |
|  | Bosc | 3.63E5 (1.43E5 - 6.20E6) | 10 |  | **3.75E5 (1.92E5 - 7.41E6)** | 20 |  | 95.5 | 150.5 , 314.5 | 0.8538 |

^a^ Bold letters highlight the median cells/g in R2 that were higher than in R1, regardless of the result of the statistical analysis.

^b^ Asterisks indicate the level of significancy: **, *P* < 0.01.

**Supplementary Table S13.** Comparison between *E. amylovora* populations in Asian pear cankers sampled in two experiment repeats, R1 (2016-2017) and R2 (2017-2018). Differences between R1 and R2 were assessed separately for each cultivar and canker harvesting period, by two-tailed Mann-Whitney *U* tests (α = 0.05).

|  |  | **R1 (2016 - 2017)** | |  | **R2 (2017 - 2018)** | |  |  |  |  |
| --- | --- | --- | --- | --- | --- | --- | --- | --- | --- | --- |
| **Canker Harvest. Period** | **Cultivar** | **Median (Q1-Q3) (Cells/g)** | **n** |  | **Median (Q1-Q3) (Cells/g)^a^** | **n** | **Comparison** | **Mann-Whitney *U* Statistic** | **Sum of Ranks (R1, R2)** | ***P value^b^*** |
| July | Hosui | 3.25E8 (6.89E7 - 5.09E8) | 13 |  | 2.62E8 (6.39E7 - 1.33E9) | 13 | R1 vs R2 | 72 | 163 , 188 | 0.5446 |
|  | Shinko | 8.44E7 (3.28E7 - 1.68E8) | 10 |  | **2.03E8 (5.71E7 - 4.92E8)** | 13 |  | 43 | 98 , 178 | 0.1862 |
|  | Yoinashi | 2.96E7 (3.31E5 - 5.71E7) | 3 |  | **9.92E7 (2.73E7 - 3.38E8)** | 16 |  | 11 | 17, 173 | 0.1713 |
| October | Hosui | 8.03E5 (5.26E5 - 1.12E6) | 8 |  | **2.36E8 (5.82E7 - 4.59E8)** | 8 | R1 vs R2 | 0 | 36 , 100 | 0.0002 (***) |
|  | Shinko | 3.65E5 (2.57E5 - 1.04E6) | 5 |  | **5.57E6 (1.53E6 - 4.55E7)** | 8 |  | 2 | 17 , 74 | 0.0062 (**) |
|  | Yoinashi | 4.13E5 (3.61E5 - 6.23E5) | 7 |  | **4.84E6 (1.27E6 - 4.21E7)** | 9 |  | 6 | 34 , 102 | 0.0052 (**) |
| January | Hosui | 7.63E5 (5.90E5 - 7.62E6) | 4 |  | **2.81E6 (9.78E5 - 4.79E6)** | 7 | R1 vs R2 | 7 | 17 , 49 | 0.2303 |
|  | Shinko | 0.00E0 (0.00E0 - 0.00E0)^c^ | 3 |  | **9.75E6 (2.44E5 - 1.77E7)** | 12 |  | 0 | 6 , 114 | 0.0044 (**) |
|  | Yoinashi | 1.67E5 (1.67E5 - 1.67E5) | 1 |  | **5.37E6 (9.01E5 - 6.10E7)** | 16 |  | NA^d^ | NA^d^ | NA^d^ |
| April | Hosui | 0.00E0 (0.00E0 - 0.00E0)^c^ | 3 |  | **1.07E6 (4.81E5 - 8.96E7)** | 3 | R1 vs R2 | 0 | 21 , 24 | 0.0119 (*) |
|  | Shinko | 0.00E0 (0.00E0 - 0.00E0)^c^ | 3 |  | **3.03E6 (3.27E5 - 6.74E7)** | 15 |  | 0 | 6 , 165 | 0.0025 (**) |
|  | Yoinashi | 1.00E5 (7.95E4 - 3.47E5) | 6 |  | **8.18E7 (1.16E6 - 2.31E8)** | 12 |  | 2 | 23 , 148 | 0.0004 (***) |

^a^ Bold letters highlight the median cells/g in R2 that were higher than in R1, regardless of the result of the statistical analysis.

^b^ Asterisks indicate the level of significancy: *, *P* < 0.05; **, *P* < 0.01; ***, *P* < 0.001.

^c^ A 0% of the cankers collected from ‘Shinko’ and/or ‘Hosui’ trees in the indicated time point were positive for *E. amylovora* detection. The absence of positives was arbitrarily substituted to perform the analysis by three replicates of 0 cell/g.

^d^ Because in the first experiment repeat (R1), only one canker from ‘Yoinashi’ trees resulted positive for *E. amylovora* detection in January*,* it was not possible to perform the comparison between R2 and R1. NA, not apply.

**Supplementary Table S14.** Associations between *E. amylovora* detection by viability dPCR and the host resistance to fire blight, the season of the year in which samples were collected and the experimental repeat (N =819).

|  |  |  |  |  | **Univariate logistic regression^a^** | |  | **Multivariable logistic regression^b^** | | |
| --- | --- | --- | --- | --- | --- | --- | --- | --- | --- | --- |
| Variable and Levels | | Total^c^ | Pos.^d^ | Neg.^e^ | OR (95% CI) | ***P* value** |  | OR (95% CI) | ***P* value** | VIF^f^ |
| Degree of resistance to fire blight | |  |  |  |  |  |  |  |  |  |
|  | Extremely susceptible (ES) | 199 | 124 | 75 | Ref.^g^ |  |  | Ref. |  |  |
|  | Highly Susceptible (HS) | 305 | 208 | 97 | 1.297 (0.8910 to 1.886) | 0.1736 |  | 1.676 (1.076 to 2.621) | **<0.0001** | 1.6 |
|  | Susceptible (S) | 213 | 185 | 28 | 3.996 (2.473 to 6.610) | **<0.0001** |  | 6.482 (3.740 to 11.53) | **<0.0001** | 1.54 |
|  | Moderately Susceptible (MS) | 101 | 87 | 14 | 3.759 (2.048 to 7.325) | **<0.0001** |  | 7.199 (3.619 to 15.14) | **<0.0001** | 1.34 |
| Canker harvesting period | |  |  |  |  |  |  |  |  |  |
|  | July (summer) | 158 | 153 | 5 | 24.43 (10.65 to 70.77) | **<0.0001** |  | 40.44 (16.83 to 120.9) | **0.0229** | 1.36 |
|  | October (fall) | 198 | 157 | 41 | 3.057 (2.001 to 4.738) | **<0.0001** |  | 4.495 (2.770 to 7.436) | **<0.0001** | 1.44 |
|  | January (winter) | 232 | 129 | 103 | Ref. |  |  | Ref. |  |  |
|  | April (spring) | 230 | 165 | 65 | 2.027 (1.380 to 2.993) | **0.0003** |  | 2.335 (1.508 to 3.649) | **0.0002** | 1.44 |
| Experimental repeat | |  |  |  |  |  |  |  |  |  |
|  | R1 (2016-2017) | 382 | 231 | 151 | Ref. |  |  | Ref. |  |  |
|  | R2 (2017-2018 / 2018-2019) | 436 | 373 | 63 | 3.87 (2.776 to 5.448) | **<0.0001** |  | 5.456 (3.705 to 8.162) | **<0.0001** | 1.0 |

^a^ Determines associations between *E. amylovora* detection with separate predictors. The provided Odds Ratio and 95% Confidence Intervals, OR (95% CI), are unadjusted. Significant *P* values (in bold letters) (α = 0.05) indicate an association between the analyzed factor and the outcome (without considering other variables).

^b^ Analyzes the effect of each variable in the outcome while accounting for the simultaneous effect of the other variables included in the model. The model provides adjusted OR (95% CI). Bold letters indicate a significant association (*P* < 0.05) between the variable level and the outcome, while accounting for the concurrent effects of the other variables.

^c-e^ Total, positive and negative number of cankers for *E. amylovora* detection, respectively.

^f^ Variance Inflation Factor, indicator of collinearity between the variables in the model.

^g^ Reference level (baseline) for result interpretation.

**Supplementary Table S15.** Pairwise comparison between logistic regression models explaining the associations between *E. amylovora* detection in pome fruit tree cankers and different predictor variables. The more complex model containing all the predictor variables (Model 8) was compared to simpler models missing one or more variables (Models 1 to 7). The table shows parameters related to model comparison and goodness-of-fit diagnostics.

| **Model no.** | **Model description** | **AICc** | **AUC** | **NPP** | **PPP** | **Tjur's R2** | **McFadden R2** | **HL test** | **LRT** |
| --- | --- | --- | --- | --- | --- | --- | --- | --- | --- |
| 1 | Outcome ~ I | 942.3 | 0.5000 | - | 73.84 | 6.99E-15 | -6.529E-15 | - | - |
| 2 | Outcome ~ I+FBSI | 900.3 | 0.6414 | - | 73.84 | 0.05538 | 0.05109 | >0.9999 | <0.0001 |
| 3 | Outcome ~ I+CHP | 847.0 | 0.7037 | - | 73.84 | 0.1061 | 0.1078 | >0.9999 | <0.0001 |
| 4 | Outcome ~ I+REP | 876.9 | 0.6616 | - | 73.84 | 0.08105 | 0.0717 | >0.9999 | <0.0001 |
| 5 | Outcome ~ I+FBSI+CHP | 793.0 | 0.7716 | 52.05 | 79.46 | 0.1794 | 0.1716 | 0.3783 | <0.0001 |
| 6 | Outcome ~ I+FBSI+REP | 830.0 | 0.7356 | 56.82 | 77.53 | 0.1481 | 0.1280 | 0.6986 | <0.0001 |
| 7 | Outcome ~ I+CHP+REP | 775.9 | 0.7798 | 63.81 | 79.38 | 0.1987 | 0.1856 | 0.9641 | <0.0001 |
| **8** | **Outcome ~ I+FBSI+CHP+REP** | **713.8** | **0.8293** | **67.72** | **83.79** | **0.2827** | **0.2581** | 0.0496 | **<0.0001** |

* Each column summarizes:

- Model number and model description. Outcome, positive *E. amylovora* detection; I, intercept; FBSI, fire blight severity index; CHP, canker harvesting period; REP, experiment repeat. Models were built using data from non-irrigated trees.

- AICc, Logistic regression model comparison (pairwise) by the corrected Akaike’s Information Criterion. The method determines how well the data support each model. The best models have the lowest AICc values. Models 1-15 were compared to the one containing all the variables (Model 16, in bold letters).

- AUC, Area Under the ROC curve. Evaluates the model’s classification performance (cutoff 0.5) in terms of sensitivity and specificity. Higher AUC values support a better classification performance of the model.

- NPP (%) and PPP (%), the model’s negative and positive prediction power, respectively. E.g., a PPP (%) = 60% indicates that 60% of the positives were correctly classified as positives by the model.

- Tjur’s R^2^ and McFadden’s R^2^ are pseudo R^2^ parameters used as indicators of the model’s goodness-of-fit (the higher their value, the better results are explained by the model). In general, the higher their value, the better fit. McFadden R^2^ between 0.2 and 0.4 represent an excellent fit ().

-HL, Hosmer-Lemeshow Test. Tests the null hypothesis that the specified model is correct. *P* values > 0.05 indicate that there is no evidence to reject the null hypothesis, i.e., the proposed model explains the results better than a model involving no effects associated with any of the variables).

- LRT, Log-likelihood Ratio test. Tests the null hypothesis that the intercept-only model fits the data best. *P* values < 0.05 indicate that there is evidence to think that the obtained data is better explained by the alternative model, i.e., the specified independent variables and interactions improve the fit of the model to the data.

**Supplementary Table S16.** Associations between *E. amylovora* detection by v-dPCR, the host resistance to fire blight and the year in which samples were collected.

|  |  |  | **Multivariable logistic regression^a^** | | |
| --- | --- | --- | --- | --- | --- |
| Sampling point | Variable and Levels | | OR (95% CI) | *P* value | VIF^b^ |
| July | Degree of resistance to fire blight | |  |  |  |
| (N=158) |  | Extremely susceptible (ES) | Ref.^c^ |  |  |
|  |  | Highly Susceptible (HS) | NA^d^ | NA | NA |
|  |  | Susceptible (S) | NA | NA | NA |
|  |  | Moderately Susceptible (MS) | NA | NA | NA |
|  | Experiment repeat | |  |  |  |
|  |  | R1 | Ref. |  |  |
|  |  | R2 | NA | NA | NA |
| October | Degree of resistance to fire blight | |  |  |  |
| (N=198) |  | Extremely susceptible (ES) | Ref. |  |  |
|  |  | Highly Susceptible (HS) | 2.186 (0.9630 to 5.066) | 0.0634 | 1.5 |
|  |  | Susceptible (S) | 17.57 (4.596 to 116.3) | **0.0003** | 1.5 |
|  |  | Moderately Susceptible (MS) | 3.529 (1.110 to 13.73) | **0.0448** | 1.3 |
|  | Experiment repeat | |  |  |  |
|  |  | R1 | Ref. |  |  |
|  |  | R2 | 3.091 (1.445 to 6.967) | **0.0046** | 1.0 |
| January | Degree of resistance to fire blight | |  |  |  |
| (N=232) |  | Extremely susceptible (ES) | Ref. |  |  |
|  |  | Highly Susceptible (HS) | 1.013 (0.4921 to 2.091) | 0.9714 | 1.6 |
|  |  | Susceptible (S) | 2.504 (1.109 to 5.814) | **0.0293** | 1.5 |
|  |  | Moderately Susceptible (MS) | 4.156 (1.498 to 12.44) | **0.0079** | 1.3 |
|  | Experiment repeat | |  |  |  |
|  |  | R1 | Ref. |  |  |
|  |  | R2 | 5.211 (2.928 to 9.532) | **<0.0001** | 1.0 |
| April | Degree of resistance to fire blight | |  |  |  |
| (N=230) |  | Extremely susceptible (ES) | Ref. |  |  |
|  |  | Highly Susceptible (HS) | 4.283 (1.797 to 10.90) | **0.0014** | 1.9 |
|  |  | Susceptible (S) | 20.28 (6.918 to 67.69) | **<0.0001** | 1.8 |
|  |  | Moderately Susceptible (MS) | 40.79 (9.325 to 295.1) | **<0.0001** | 1.5 |
|  | Experiment repeat | |  |  |  |
|  |  | R1 | Ref. |  |  |
|  |  | R2 | 8.502 (4.009 to 19.63) | **<0.0001** | 1.0 |

^a^ Analyzes the concurrent effect of each predictor variable in the outcome after adjusting for the effect of the other variables included in the model (in this case, the experiment repeat). The model provides adjusted OR (95% CI). Bold letters (*P* < 0.05) show a significant association between the variable level and the outcome after accounting for simultaneous effects of the other variables.

^b^ Variance Inflation Factor, indicator of collinearity between the variables included in the model.

^c^ Reference (baseline) level for result interpretation.

^d^ NA, not apply. Because there is a perfect separation of values, i.e., all the values in 3 out of the 4 assayed variable levels are the same in the two experiment repeats (all positives). Under these conditions, the software’s algorithm cannot calculate the parameters. The chances of detecting *E. amylovora* are the same, regardless of the experiment repeat or the host’s resistance to fire blight.

# Supplementary Figures


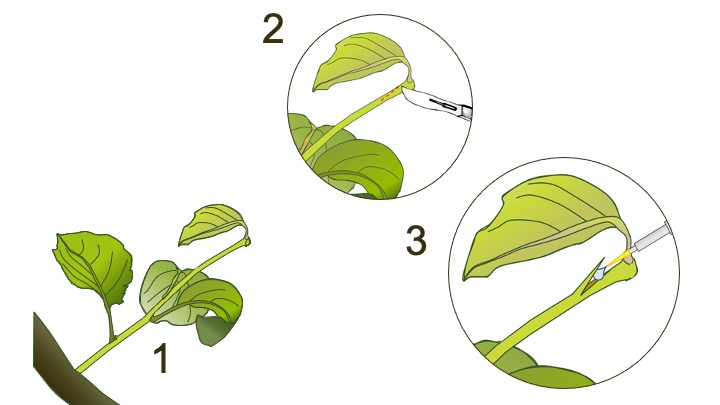


**Supplementary Figure S1. Shoot inoculation method used in this study.** Inoculations were performed on newly developed shoots from perennial branches, before terminal bud set, when shoot lengths reached between 5 and 7.5 inches (13 - 19 cm) (1). To standardize the inoculum dose per shoot, we used a sterile scalpel to cut a diagonal section of the shoot below the tip, to create a sleeve cut on the stem (2). Then, using a micropipette, 40 μL of an *E. amylovora* suspension at 10^9^ CFU/mL were placed between the exposed tissues of the shoot after the cut, and the cut sleeve with a micropipette, allowing the plant tissue to absorb the inoculum droplet (ca. 4 x 10^7^ CFU/shoot) within the next minutes (3).


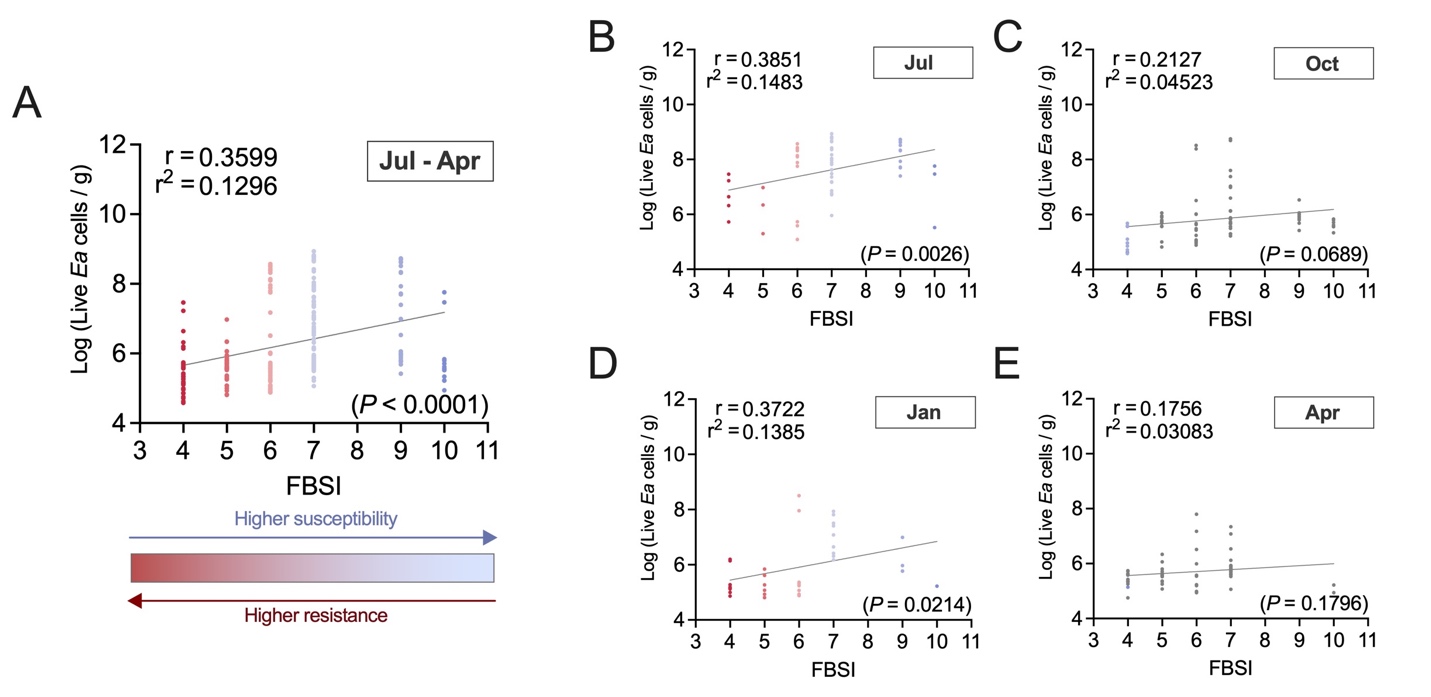


**Supplementary Figure S2. Correlation between the fire blight resistance of the host plants included in this study and the *E. amylovora* population sizes in cankers collected in the first experiment repeat.** Each chart shows the Pearson coefficient of correlation (r), coefficient of determination (r^2^), regression line and *P* value for the statistical testing. Dots correspond to *E. amylovora* population data from individual cankers, colored in function of the host’s resistance/susceptibility to fire blight estimated with the fire blight severity index (FBSI). Only graphs where the correlation was significant are colored. Chart A describes the overall correlation analysis using samples collected throughout the first experiment repeat (N = 231), while charts B, C, D and E illustrate separate data from samples collected in July (N = 59), October (N = 74), January (N = 38) and April (N = 60), respectively.


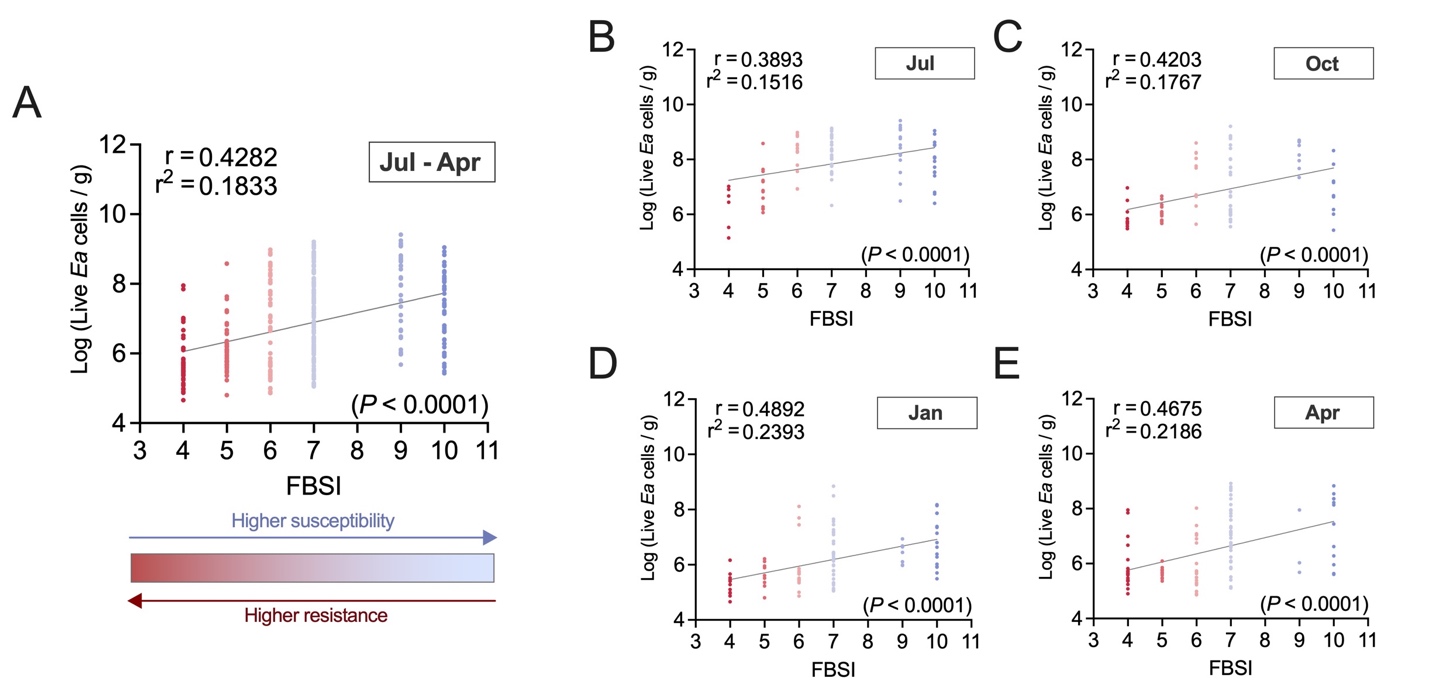


**Supplementary Figure S3. Correlation between the fire blight resistance of the host plants included in this study and the *E. amylovora* population sizes in cankers collected in the second experiment repeat.** Each chart shows the Pearson coefficient of correlation (r), coefficient of determination (r^2^), regression line and *P* value for the statistical testing. Dots correspond to *E. amylovora* population data from individual cankers, colored in function of the host’s resistance/susceptibility to fire blight estimated with the fire blight severity index (FBSI). Chart A describes the overall correlation analysis using samples collected throughout the second experiment repeat (N = 373), while charts B, C, D and E illustrate separate data from samples collected in July (N = 94), October (N = 183), January (N = 91) and April (N = 105), respectively.


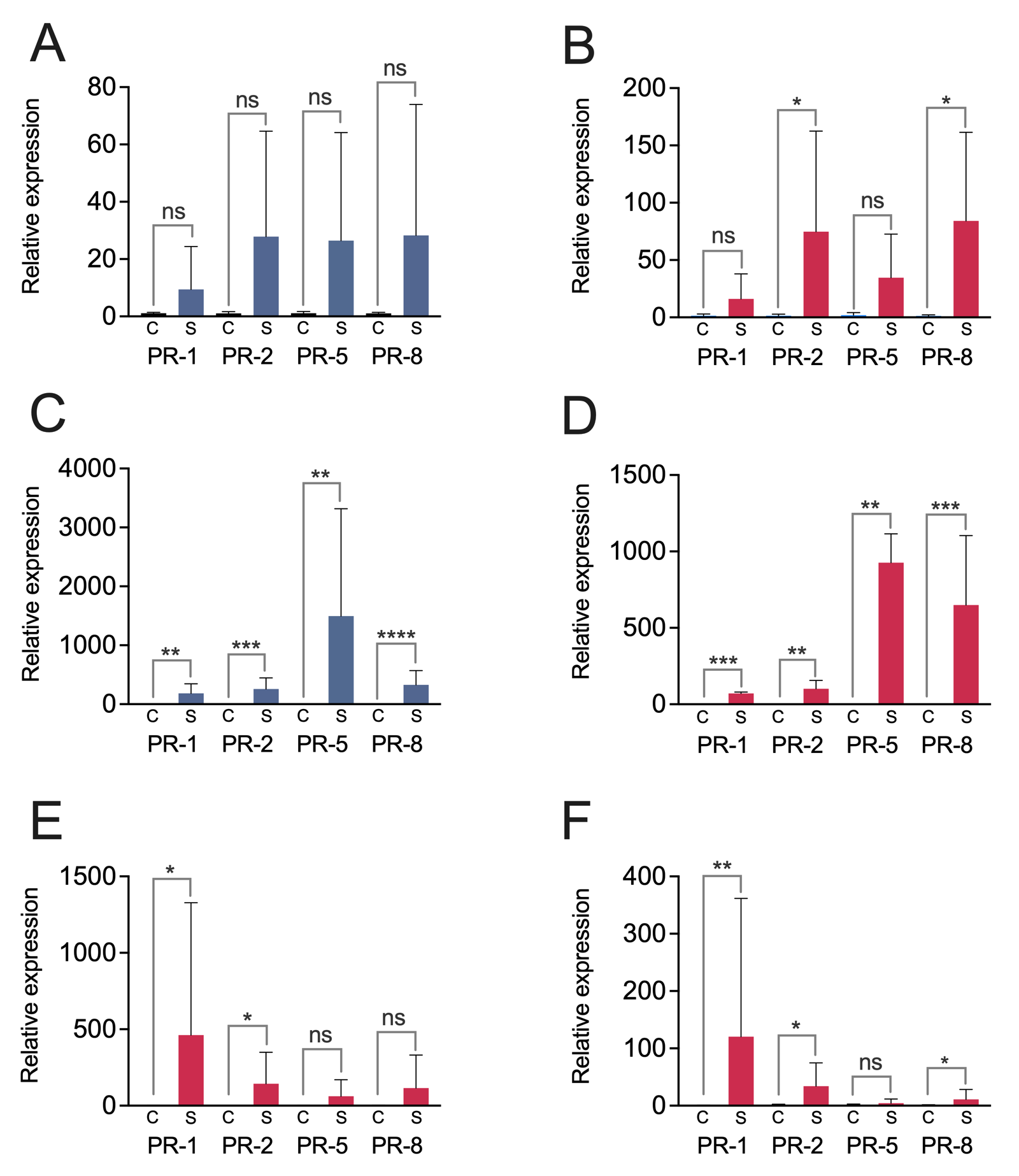


**Supplementary Figure S4. Relative expression levels of *PR* genes in cankers of *E. amylovora* host plants with different degrees of resistance to fire blight.** We analyzed cankers from the apple cultivars ‘Cortland’ (highly susceptible) (A, B) and ‘Honeycrisp’ (moderately susceptible) (C, D), and the Asian pear cultivars ‘Hosui’ (extremely susceptible) (E) and ‘Shinko’ (highly susceptible) (F). Blue (A, C) and red columns (B, D-F) show results obtained with cankers from irrigated and non-irrigated trees, respectively. Columns represent average relative expression values from one assay performed in 2016-2017, calculated by the 2^-ΔΔCt^ method, normalizing data to the reference actin gene. The error bars illustrate the SD. Asterisks indicate statistically significant differences between the relative expression of the indicated *PR* gene in the control (C) and in the canker samples (S), assessed by two-tailed unpaired *t*-tests, *, *P* < 0.05; **, *P* < 0.01; ***, *P* < 0.001; ****, *P* < 0.0001; ns, not significant (*P* > 0.05).


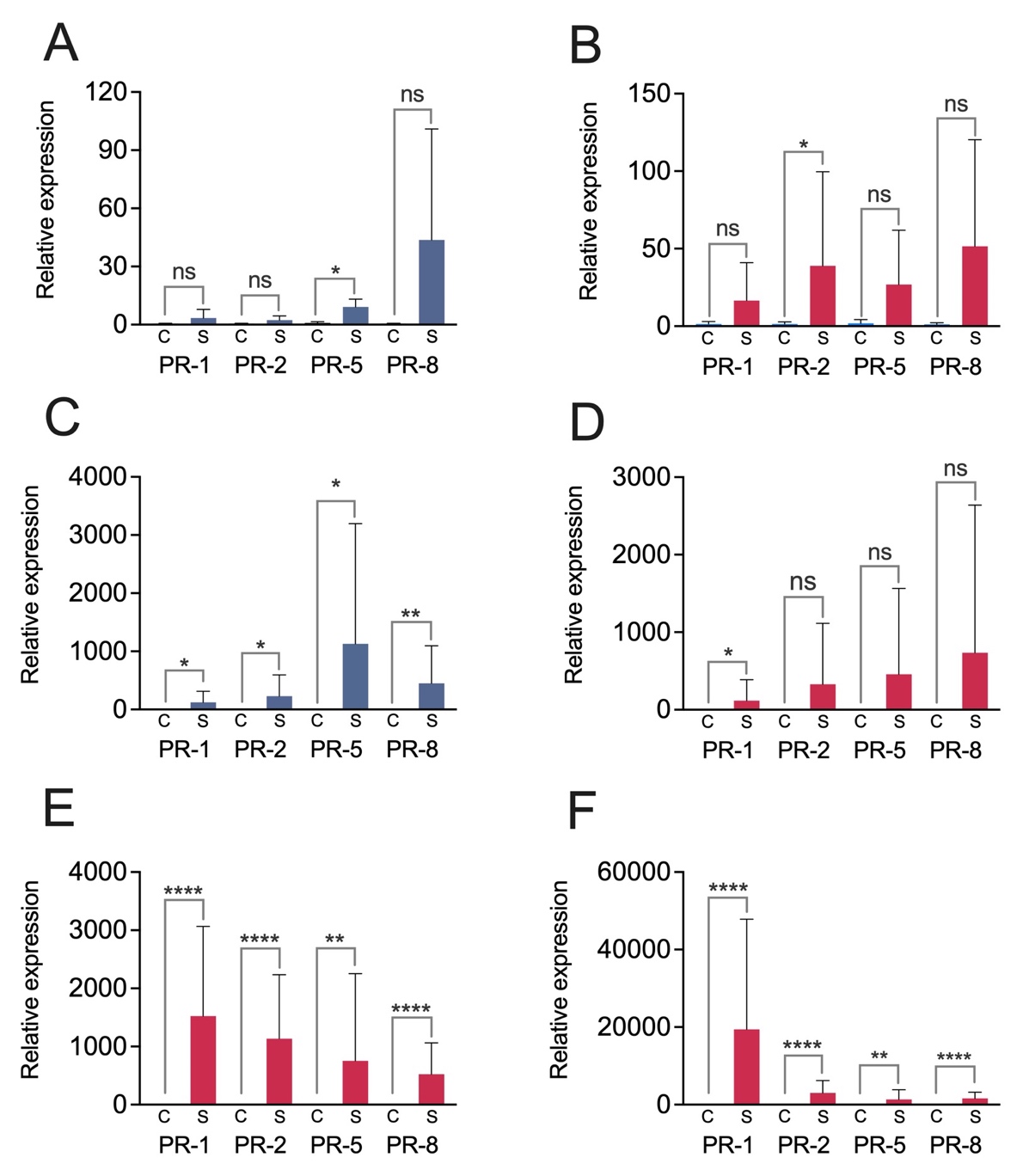


**Supplementary Figure S5. Relative expression levels of *PR* genes in cankers of *E. amylovora* host plants with different degrees of resistance to fire blight.** We analyzed cankers from the apple cultivars ‘Cortland’ (highly susceptible) (A, B) and ‘Honeycrisp’ (moderately susceptible) (C, D), and the Asian pear cultivars ‘Hosui’ (extremely susceptible) (E) and ‘Shinko’ (highly susceptible) (F). Blue (A, C) and red columns (B, D-F) show results obtained with cankers from irrigated and non-irrigated trees, respectively. Columns represent average relative expression values from an assay performed in 2018-2019 (apple) and 2017-2018 (Asian pear), calculated by the 2^-ΔΔCt^ method, normalizing data to the reference actin gene. The error bars illustrate the SD. Asterisks indicate statistically significant differences between the relative expression of the indicated *PR* gene in the control (C) and in the canker samples (S), assessed by two-tailed unpaired *t*-tests, *, *P* < 0.05; **, *P* < 0.01; ***, *P* < 0.001; ****, *P* < 0.0001; ns, not significant (*P* > 0.05).
